# Generation of a MyoD knock-in reporter mouse line to study muscle stem cell dynamics and heterogeneity

**DOI:** 10.1016/j.isci.2023.106592

**Published:** 2023-04-08

**Authors:** Ryo Fujita, Seiya Mizuno, Taketaro Sadahiro, Takuto Hayashi, Takehito Sugasawa, Fumihiro Sugiyama, Yusuke Ono, Satoru Takahashi, Masaki Ieda

**Affiliations:** 1 Division of Regenerative Medicine, Transborder Medical Research Center, Institute of Medicine, University of Tsukuba, Ibaraki 305-8575, Japan; 2 Department of Cardiology, Institute of Medicine, University of Tsukuba, Ibaraki 305-8575, Japan; 3 Laboratory Animal Resource Center, Transborder Medical Research Center, Institute of Medicine, University of Tsukuba, Ibaraki 305-8575, Japan; 4 Department of Anatomy and Embryology, Institute of Medicine, University of Tsukuba, Ibaraki 305-8575, Japan; 5 Laboratory of Clinical Examination and Sports Medicine, Department of Clinical Medicine, Institute of Medicine, University of Tsukuba, Ibaraki 305-8575, Japan; 6 Department of Muscle Development and Regeneration, Institute of Molecular Embryology and Genetics, Kumamoto University, Kumamoto 860-0811, Japan

**Keywords:** Cell biology, Molecular biology, Stem cells research

## Abstract

Myoblast determination protein 1 (MyoD) dynamics define the activation status of muscle stem cells (MuSCs), aiding in muscle tissue regeneration after injury. However, the lack of experimental platforms to monitor MyoD dynamics *in vitro* and *in vivo* has hampered the investigation of fate determination and heterogeneity of MuSCs. Herein, we report a MyoD knock-in (MyoD-KI) reporter mouse expressing tdTomato at the endogenous *MyoD* locus. Expression of tdTomato in MyoD-KI mice recapitulated the endogenous MyoD expression dynamics *in vitro* and during the early phase of regeneration *in vivo*. Additionally, we showed that tdTomato fluorescence intensity defines MuSC activation status without immunostaining. Based on these features, we developed a high-throughput screening system to assess the effects of drugs on the behavior of MuSCs *in vitro*. Thus, MyoD-KI mice are an invaluable resource for studying the dynamics of MuSCs, including their fate decisions and heterogeneity, and for drug screening in stem cell therapy.

## Highlights

• MyoD knock-in mice expressing tdTomato was generated by CRISPR-Cas9
• MyoD-tdTomato recapitulates endogenous MYOD expression during early myogenesis
• MyoD-tdTomato fluorescence intensity defines the activation status of MuSCs
• MyoD-KI mice aid in drug screening that regulate the fate of MuSCs *in vitro*

## Graphical abstract

**Figure:**
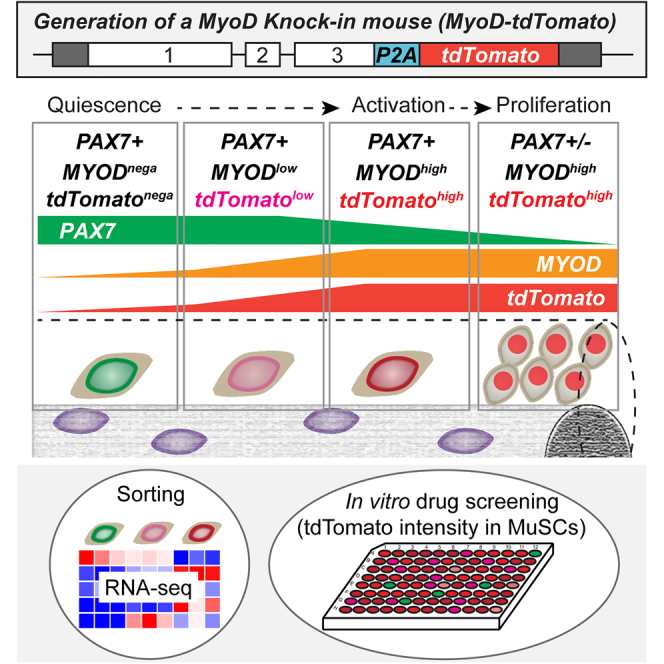

## Introduction

Skeletal muscles display a remarkable regenerative capacity, which depends on skeletal muscle stem cells (MuSCs), also known as satellite cells, found around the myofibers and underneath the basal lamina.^1^ MuSCs represent myogenic progenitors that supply nuclei to growing myofibers during development and regeneration.^2^ In adult muscles, MuSCs enter a mitotically quiescent state. However, in response to injury, they are activated and enter the cell cycle to rapidly expand myogenic progenitors that either differentiate into new myofibers or undergo self-renewal for lifelong maintenance of the MuSC pool.^3^^,^^4^

MuSC dynamics are regulated by multiple transcription factors sequentially expressed during myogenesis. Quiescent MuSCs express members of the paired homeodomain family of transcriptional factors paired box protein *Pax7,*^5^ and a small subset of quiescent MuSCs express *Pax3*, the paralog of *Pax7.*^6^^,^^7^^,^^8^ Myogenic regulatory factors (MRFs) comprising myoblast determination protein 1 (*Myod1, MyoD*) and myogenic factor 5 (*Myf5*) are fundamental regulators of skeletal muscle lineage determination and development and are also induced in activated MuSCs during regeneration in adult mice. Interestingly, quiescent MuSCs are primed to rapidly activate the myogenic program while remaining quiescent.^9^^,^^10^^,^^11^ These unique paradoxical features are mainly achieved by the translational suppression of *MyoD* and *Myf5* mRNAs with microRNAs and RNA-binding proteins (RBPs). *Myf5* transcripts are repressed by miR-31 and fragile X mental retardation protein associated with cytoplasmic RNA granules.^9^^,^^12^^,^^13^ *MyoD* transcripts are repressed by the activity of RBPs, such as Zfp36 and Staufen1 (*Stau1*), through their 3′-UTR.^14^^,^^15^ Upon MuSC activation, *MyoD* and *Myf5* transcripts are translated to initiate the myogenic program by relieving this translational block.

The functions of *MyoD* and *Myf5* as established transcriptional factors for myogenetic lineage specification are partially redundant and contribute to establishment of the myogenic progenitors during embryogenesis.^16^^,^^17^^,^^18^^,^^19^^,^^20^^,^^21^ In addition, *MyoD* plays a unique role in adult muscle regeneration, as reported by the delayed onset of myogenic differentiation and increased propensity for self-renewal in *MyoD* knockout (KO) mice, suggesting that MYOD regulates the balance between self-renewal and differentiation during regeneration.^22^^,^^23^^,^^24^^,^^25^ Furthermore, *MyoD* KO mice on a mdx background, a model of Duchenne muscular dystrophy, shows poor survival and die around the age of 12 months^22^

MYOD has also been used as an activation marker for MuSCs during myogenic development and regeneration. The combination of MYOD and PAX7 immunostaining is frequently used to define the divergent fates of MuSCs during myogenesis and culture. Quiescent MuSCs are PAX7 positive (+) but MYOD-negative (−), whereas activated MuSCs co-express PAX7 and MYOD. Most activated MuSCs then proliferate, downregulate PAX7, and differentiate by expressing MYOGENIN, a member of the MRFs. In contrast, a small number of PAX7 (+), MYOD (+) activated MuSCs downregulate MYOD while maintaining PAX7 to return to the state resembling quiescence, which represents “reserve stem cell”.^26^^,^^27^^,^^28^ The mechanism by which activated MuSCs determine cell fate to proceed or return to the myogenic program remains unclear. Particularly, although MuSCs are maintained in an undifferentiated state by manipulating substrate elasticity or p38 Mitogen-activated Protein Kinase (MAPK) activity,^29^^,^^30^^,^^31^ stimulating and maintaining the reserve stem cell population, a defining feature of stem cells *in vitro* and *in vivo*, remains a major challenge in muscle stem cell research.

Since *Pax7* and *MyoD* are transcription factors, a major challenge is the lack of an efficient tool to isolate and analyze immunolabeled MuSC populations at different myogenic stages *in vitro* and *in vivo*. Recently, two Pax7-knock-in (KI) reporter mouse lines have been generated.^32^^,^^33^ The *Pax7*-KI mouse line is a valuable tool for isolating quiescent MuSCs and studying a heterogeneous population of quiescent MuSCs based on PAX7 expression levels. However, several limitations of the Pax7-KI mouse line hinder our understanding of the transition from quiescence to activation and vice versa. MYOD expression pattern reflects the myogenic stage of MuSCs more accurately during myogenic progression. Moreover, accumulating evidence indicates that MuSCs are functionally heterogeneous populations, including rapidly dividing majorities and slowly dividing minorities.^34^^,^^35^ Therefore, recapitulating endogenous MYOD expression in MuSCs with a fluorescent reporter is urgently required to distinguish and study the divergent phenotypes of MuSCs during myogenesis.

CRISPR-Cas9 is a potent tool for precise genome editing, and we used it to generate a MyoD-KI mouse line expressing tdTomato from the *MyoD* locus, without affecting the endogenous MYOD functions. Here, we assessed the MyoD-KI tdTomato reporter mirrors endogenous expression patterns *in vitro* and *in vivo*. Furthermore, we analyzed the tdTomato fluorescence intensity of MuSCs isolated from MyoD-KI mice in correlation with the activation status of MuSCs in culture, as evidenced by protein and RNA-sequencing analyses. Finally, using the features of MyoD-KI tdTomato fluorescence in cultured MuSCs, we aimed to establish an unbiased and high-throughput screening system to analyze the effect of compounds on MuSCs based on tdTomato fluorescence intensity. Overall, our MyoD-KI mouse line would open up an opportunity to elucidate the molecular mechanisms of MuSC dynamics, including their heterogeneity and fate determination mechanism, and to develop a potential drug target for MuSCs for regenerative medicine.

## Results

### Generation of a MyoD-KI mouse line by CRISPR-Cas9

**Figure 1:**
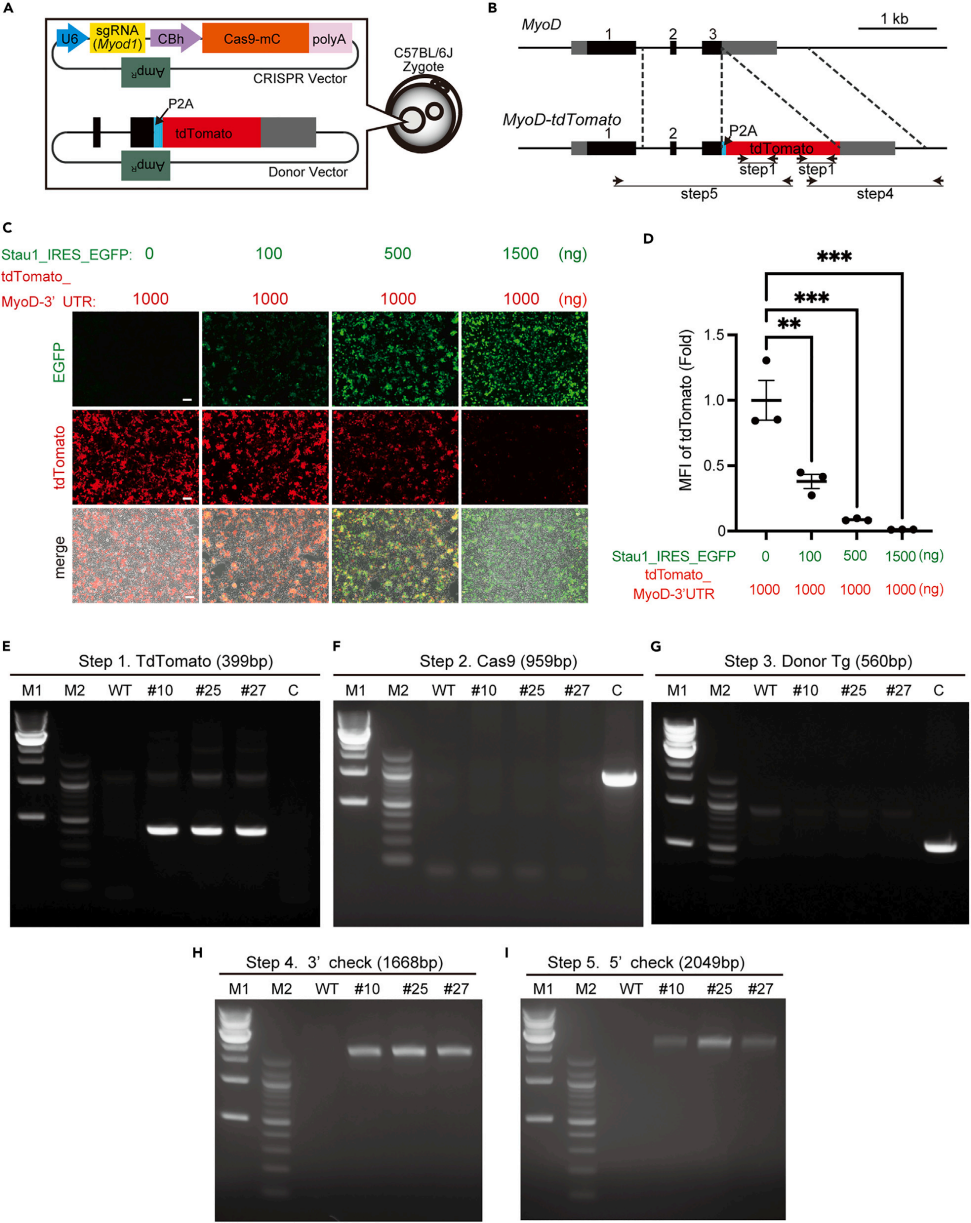
MyoD-tdTomato-knock-in (KI) mice are generated by CRISPR-Cas9 (A) Design of CRISPR and the donor vector microinjected into the C57BL/6J zygote. (B) Schematic diagram of KI of tdTomato to the *MyoD* locus. (C) Representative fluorescence images of HEK293T cells co-transfected with pcDNA3.1_Staufen1_IRES_EGFP and pcDNA3.1_tdTomato_3′-UTR MyoD expression vectors. Scale bar: 50 μm. (D) Mean fluorescence intensity (MFI) of tdTomato in C, determined using flow cytometry analysis (n = 3). (E–I) Genomic DNA analysis of F0 *MyoD*^*KI/+*^ or WT mice tails using PCR. The primer sets used for step 1, 4, and 5 are depicted in (B). Image showing WT (lane 3) and 3 different lines of F0 male MyoD^KI/+^ mice (lane 4–6). Lane 7 is Cas9-amp control to assess the random integration. Each expected PCR amplicon size is indicated in parentheses. M1, 1 kb DNA ladder; M2, 100 bp DNA ladder. All data are represented as the mean ± standard error of the mean (s.e.m.) ∗∗p < 0.01, ∗∗∗p < 0.001.

To generate MyoD-KI (*MyoD*^*KI/+*^) mice using the CRISPR-Cas9 system, a single guide RNA (sgRNA) was designed to target the region of the stop codon of *MyoD* (Figure 1A). sgRNA and Cas9 were inserted into the *px330-mC* plasmid driven by the U6 and CBh promoter, respectively. For the KI donor DNA vector, the tdTomato sequence, preceded by a P2A peptide sequence, was cloned into the *px330-mC* plasmid (Figure 1A). The KI of the donor DNA vector consisted of two homology arms corresponding to the end of exon 3 and the start of the 3′-UTR of *MyoD* sequence (Figures 1A and 1B). Microinjection of these vectors into the pronucleus of the C57BL/6J zygote resulted in the generation of the *MyoD-KI* locus via homologous recombination (Figure 1B). The binding of *Stau1* to the 3′-UTR of *MyoD* represses the protein translation of *MyoD.*^15^ Therefore, before generating the F0 *MyoD*^*KI/+*^ mice, we confirmed if the insertion of the *tdTomato* sequence immediately before the 3′-UTR of *MyoD* affects its post-transcriptional regulation by Staufen1 *in vitro*. To determine Staufen1 activity on the tdTomato_3′-UTR of *MyoD*, an expression vector expressing tdTomato_3′-UTR of the *MyoD* sequence under the Cytomegalovirus (CMV) promoter was created, and the plasmid was co-transfected with the Stau1_IRES_EGFP expression vector into the HEK293T cell line (Figure 1C). The tdTomato fluorescence decreased markedly as the expression of Stau1-EGFP increased (Figures 1C and 1D), suggesting that the 3′-UTR-mediated translational regulation of Staufen1 was not disrupted by the insertion of the tdTomato sequence into the *MyoD* 3′-UTR *in vitro.*

We initially screened for the presence of tdTomato (399bp) in the genomic DNA of 74 newborn mice of the F0 generation by using PCR with *tdTomato*-specific primer pairs (Figure 1E). Three male F0 founders (#10, 25, and 27) carrying the *tdTomato* sequence on their genome were further subjected to PCR screening with Cas9- (959 bp) and ampicillin-specific primer pairs (560 bp) to confirm that it was not the result of random integrations (Figures 1F and 1G). Furthermore, precise KI of the *tdTomato* sequence at the *MyoD* locus was verified by two additional PCR screenings using the primers listed in Figure 1B. The expected amplicons were confirmed in all three F0 founders (Figures 1H and 1I). Male F0 founders (*MyoD*^*KI/+*^) were mated with female C57BL/6J mice to generate heterozygous KI offspring for subsequent analyses. PCR screening was repeated on the F1 offspring for further confirmation (Figure S1).

### MyoD-tdTomato fluorescence patterns partially mirror endogenous MYOD protein expression *in vivo*

**Figure 2:**
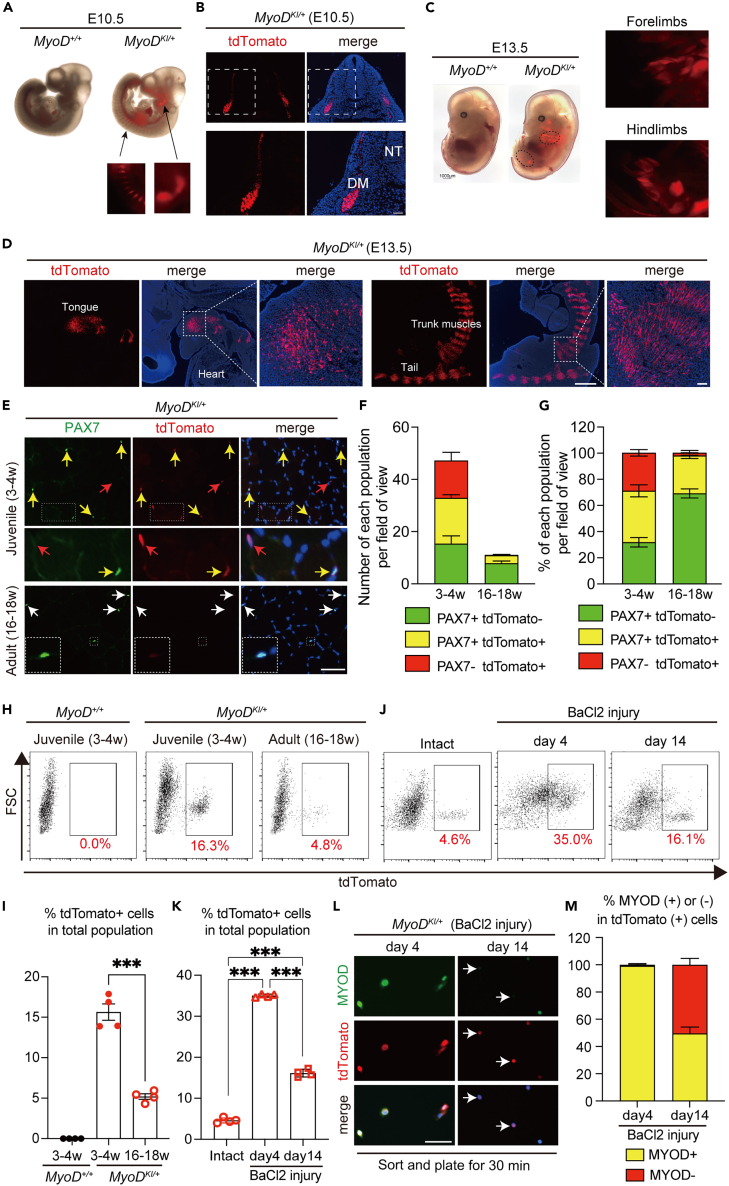
*MyoD*^*KI/+*^ mice recapitulate endogenous MYOD expression dynamics *in vivo* (A) Endogenous tdTomato fluorescence from *MyoD*^*KI/+*^ embryos at E10.5. The insets at E10.5 indicate endogenous tdTomato expression in interlimb somites and the branchial arches. (B) Transverse section of *MyoD*^*KI/+*^ embryos at E10.5 showing tdTomato expression in the dermomyotome region. Nuclei stained with DAPI. The area in the white dotted boxes is shown at a higher magnification. NT, neural tube. Scale bar: 50 μm. (C) Endogenous tdTomato fluorescence from *MyoD*^*KI/+*^ embryos at E13.5. The insets at E13.5 indicate endogenous tdTomato expression in forelimbs and hindlimbs. (D) Sagittal section of *MyoD*^*KI/+*^ embryos at E13.5. Nuclei stained with DAPI. The area in white dotted boxes is shown at higher magnification. Scale bar: 500 μm at the lower magnification, 50 μm at the higher magnification. (E) Immunofluorescence analysis using PAX7 (green) and tdTomato (red) antibodies on uninjured TA muscle cross-sections from 3-4- and 16-18-week-old male *MyoD*^*KI/+*^ mice. Yellow arrowheads indicate PAX7 (+) and tdTomato (+) muscle stem cells (MuSCs). White arrowheads indicate PAX7 (+) and tdTomato (−) MuSCs. (F and G) Quantification of numbers (F) or proportion (G) of PAX7 (+), tdTomato (−) (green), PAX7 (+), tdTomato (+) (yellow) and PAX7 (−), tdTomato (+) (red) cells in (E). (n = 4–5 mice/group). (H) Representative flow cytometry plot of the mononuclear cell fraction isolated from limb muscles of 3–4- and 16–18-week-old *MyoD*^*KI/+*^ mice. As negative control, 3–4-week-old male *MyoD*^*+/+*^(WT) mice is shown. (I) Percentage of tdTomato (+) cells at the different ages, as shown in H (n = 4 mice/group). (J) Representative flow cytometry plot of the mononuclear cell fraction isolated from limb muscles of 16–18-week-old male *MyoD*^*KI/+*^ mice at days 4 and 14 following BaCl_2_-induced injury. As a negative control, 16–18-week-old male *MyoD*^*KI/+*^ mice without injury (Intact) is shown. (K) Percentage of tdTomato (+) cells at the different time point following BaCl_2_-induce injury, as shown in J (n = 4 mice/group). (L) tdTomato (+) cells isolated from male *MyoD*^*KI/+*^ mice at day 4 and day 14 following BaCl_2_-induce injury using flow cytometer, and these cells are re-plated for 30 min to perform immunofluorescence analysis with antibodies against MYOD (green) and tdTomato (red). Scale bar: 50 μm. (M) The proportion of MYOD (+) or MYOD (−) cells in sorted tdTomato (+) cells in L (n = 4 independent experiments). All data are represented as the mean ± s.e.m. ∗∗∗p < 0.001, ns, not significant.

First, we evaluated the endogenous tdTomato fluorescence in *MyoD*^*KI/+*^ embryos at E10.5 and E13.5 (Figures 2A–2D). Consistent with a previous result of whole-mount *in situ* hybridization of *MyoD* mRNA at E10.5,^36^ tdTomato fluorescence was observed in the hypaxial myotome of interlimb somites, forelimb bud, and brachial arches of E10.5 *MyoD*^*KI/+*^ embryos (Figure 2B). Additionally, tdTomato fluorescence was detected in the trunk, limbs, and craniofacial muscles at E13.5 of whole *MyoD*^*KI/+*^ embryos (Figure 2C), which also represents a pattern similar to the endogenous *MyoD* mRNA expression, as described previously.^21^ The sagittal section at E13.5 of *MyoD*^*KI/+*^embryo showed that tdTomato (+) cells were clearly localized at the level of tongue, tail, and trunk muscles (Figure 2D).

To determine whether tdTomato fluorescence also recapitulates endogenous MYOD expression dynamics in adult MuSCs, we analyzed cross-sections of tibialis anterior (TA) muscles from 3-4- (juvenile) and 16-18-week-old (adult) *MyoD*^*KI/+*^ mice with antibodies against PAX7 and tdTomato. The TA muscle of juvenile *MyoD*^*KI/+*^ mice contained a significantly higher number of myogenic cells than adult *MyoD*^*KI/+*^ mice (Figures 2E and 2F). Further analysis of the myogenic cells in the TA muscles from juvenile *MyoD*^*KI/+*^ mice show that an average of 31% of PAX7 (+) tdTomato (−), 39% of PAX7 (+) tdTomato (+), and 30% of PAX7 (−) tdTomato (+) (Figure 2G). The analysis of single extensor digitorum longus (EDL) myofibers from juvenile *MyoD*^*KI/+*^ mice also demonstrated a similar fraction of this population (Figures S2A and S2B), thereby reflecting their activated status.^37^^,^^38^^,^^39^ In contrast, in the TA muscles from adult *MyoD*^*KI/+*^ mice, at a time point when PAX7(+) MuSCs reached quiescence,^37^^,^^38^ the myogenic cells were 70% of PAX7 (+) tdTomato (−), 28% of PAX7 (+) tdTomato (+), and 2% of PAX7 (−) tdTomato (+) cells (Figure 2G). Noteworthily, tdTomato fluorescence could not be detected in the myofibers of the TA sections from both juvenile and adult *MyoD*^*KI/+*^ mice (Figure 2E).

Next, to determine whether MuSCs expressing tdTomato can be quantified by flow cytometry, we isolated the mononuclear population from the hindlimb muscles of juvenile and adult *MyoD*^*KI/+*^ mice. Consistent with the analysis of TA muscle cross-sections with antibodies against PAX7 and tdTomato (Figures 2E–2G), 16.3% of tdTomato (+) mononuclear cells were observed in juvenile *MyoD*^*KI/+*^ mice, but this fraction was significantly decreased to 4.8% in adult *MyoD*^*KI/+*^ mice (Figures 2H and 2I). To demonstrate the tdTomato (+) cells detected by flow cytometry mirroring the endogenous MYOD expression, we sorted tdTomato (+) from the hindlimb muscles of juvenile *MyoD*^*KI/+*^ mice and immunolabelled the cell with antibodies against MYOD and tdTomato *ex vivo.* An average 80% of tdTomato (+) cells were positive for MYOD (+) (Figures S2C and S2D).

To further investigate whether tdTomato followed the expression dynamics of MYOD in the context of acute muscle injury, we injected barium chloride (BaCl_2_) into the TA muscles of adult *MyoD*^*KI/+*^ *mice*.^40^ Upon acute muscle injury, strong tdTomato signals were observed in the regenerating myofibers with central nuclei at day 4 post-injury (Figure S3A). However, at day 14 post-injury, the tdTomato fluorescence in the regenerating myofibers disappeared and reached a level similar to the wild-type (WT) background (Figure S3A), except for the mononuclear cells associated with the myofibers (Figure S3B) and small myofibers (Figure S3C).

Next, we analyzed the tdTomato (+) mononuclear fraction by flow cytometry at days 4 and 14 after injury (Figures 2J and 2K). We observed that the tdTomato (+) mononuclear fraction rapidly increased at day 4 post-injury but decreased by day 14 after injury (Figures 2J and 2K). Next, to determine whether the expanded tdTomato (+) cells induced by the injury also recapitulate endogenous MYOD expression dynamics, the tdTomato (+) cells from the damaged TA muscles of adult *MyoD*^*KI/+*^ mice at days 4 and 14 post-injury were sorted and immediately stained with antibodies against PAX7 or MYOD, and tdTomato ex *vivo* (Figures 2L and S4A–S4C). First, we found that, an average of 56% tdTonato (+) cells were negative for PAX7 at day 4 and the fraction significantly reduced at day 14 (Figure S4D). More importantly, we confirmed that an average of 99% tdTomato (+) cells were MYOD (+) at day 4 post-injury (Figures 2L–2M). However, only an average of 49% tdTomato (+) were immunolabelled with MYOD antibody (Figures 2L–2M), suggesting that the tdTomato protein remains longer than endogenous MYOD protein.

### MyoD-tdTomato fluorescence intensity detected in activated MuSCs recapitulates endogenous MYOD protein levels *in vitro*

**Figure 3:**
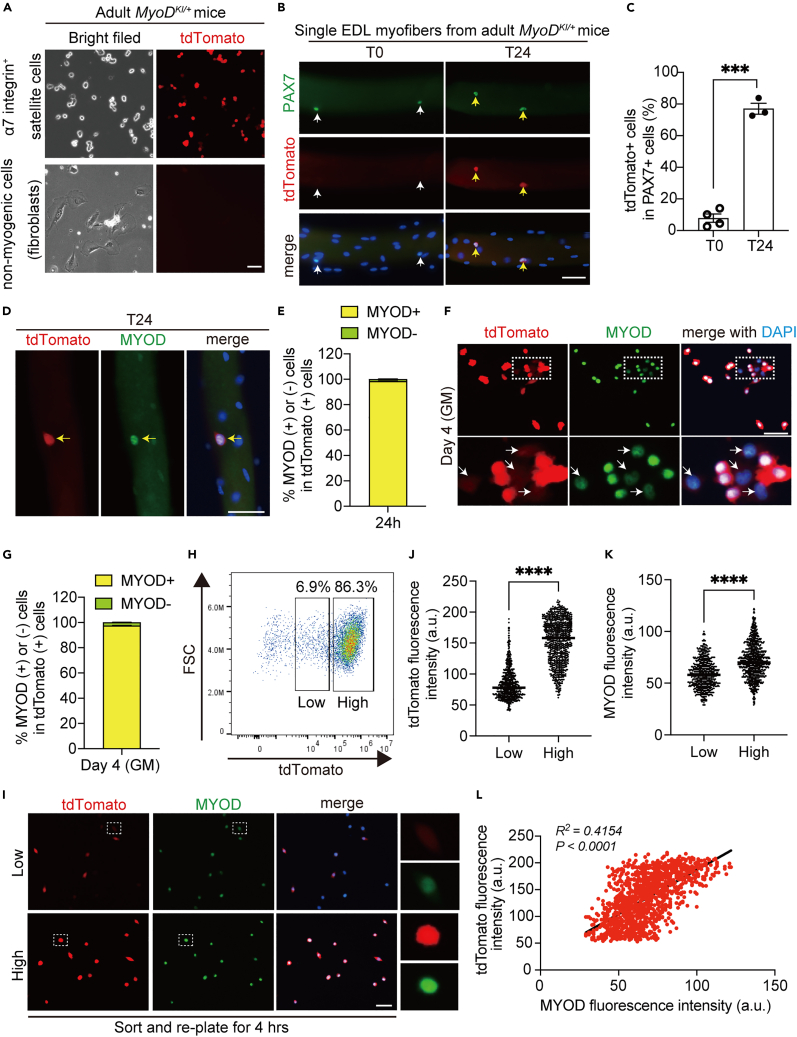
*MyoD*^*KI/+*^ mice recapitulate endogenous MYOD expression dynamics *in vitro* (A) Representative fluorescence images of magnetic cell sorting (MACS)-isolated α7-integin (+) MuSCs and α7-integin (−) non-myogenic cells. Scale bar: 50 μm. (B) Immunofluorescence of PAX7 (green) and tdTomato on freshly isolated (T0) and cultured single extensor digitorum longus (EDL) myofibers (T24) from 15 to 16-week-old *MyoD*^*KI/*+^ mice. Nuclei stained with DAPI. White arrows indicate PAX7 (+) and tdTomato (−) cells, yellow arrows indicate PAX7 (+) and tdTomato (+) cells on EDL myofibers. Scale bar: 50 μm. (C) The proportion of tdTomato (+) cells in PAX7 (+) cells on EDL myofibers in (B). (D) Immunofluorescence of MYOD (green) and tdTomato on single EDL myofibers cultured for 24 h (T24) from 15 to 16-week-old male *MyoD*^*KI/*+^ mice. Nuclei stained with DAPI. Yellow arrows indicate MYOD (+) and tdTomato (+) cells on the EDL myofibers. Scale bar: 50 μm. (E) The proportion of MYOD (+) or MYOD (−) cells in tdTomato (+) cells in D (n = 4 independent experiments). (F) Immunofluorescence against MYOD (green) and tdTomato (red) after 4-day culture of MACS-isolated MuSCs. Nuclei stained with DAPI. The area in white dotted boxes is shown at higher magnification. White arrows indicate MYOD low (+) and tdTomato low (+) cells. (G) The proportion of MYOD (+) or MYOD (−) cells in tdTomato (+) shown in (F). (H) Representative flow cytometry plot of cultured MuSCs isolated from *MyoD*^*KI/+*^ mice. The gate is defined based on the MuSC from WT mice. (I) Cultured MuSCs isolated from *MyoD*^*KI/+*^ mice sorted by flow cytometer into MyoD-tdTomato^low^ and MyoD-tdTomato^high^ populations, and these cells are re-plated for 4 h to perform immunofluorescence analysis with antibodies against MYOD (green) and tdTomato (red) (n = 4 mice). The area in white dotted boxes is shown at a higher magnification. (J and K) Quantification of tdTomato (J) and MYOD (K) fluorescence intensity in the sorted each cell in (I). (L) Correlation between tdTomato and MYOD fluorescent intensity in the sorted each cell in I. 1320 cells were analyzed. A linear model regression was calculated, and the determinant coefficient (R^2^) and p value are shown in the plot. All data are represented as the mean ± s.e.m. ∗∗∗p < 0.001.

To determine whether tdTomato fluorescence also recapitulated MYOD dynamics in *in vitro* and *ex vivo* cultures of MuSCs, we first used magnetic cell sorting (MACS) with α7-integrin microbeads to enrich MuSCs and non-myogenic progenitors from limb muscles of adult *MyoD*^*KI/+*^ mice (Figure 3A). After 4-day of culture, strong tdTomato fluorescence was exclusively observed in α7-integrin (+) MuSCs, but not in fibroblast-like non-myogenic cells (Figure 3A), suggesting that tdTomato is only expressed in myogenic progenitors. To further confirm tdTomato expression pattern, single extensor digitorum longus (EDL) myofibers were analyzed by immunohistochemistry. Single EDL myofiber from adult *MyoD*^*KI/+*^ mice stained with MYOD antibodies revealed the absence of MYOD signals in the myonuclei, and the tdTomato fluorescence was observed only faintly when compared to those from adult *MyoD*^*+/+*^ mice (Figure S5A). The analysis of single EDL myofibers isolated from adult *MyoD*^*KI/+*^ mice without culture exhibited only an average of 7.8% of tdTomato (+) in PAX7 (+) MuSCs (Figures 3B and 3C). After 24 h of culture, tdTomato (+) was rapidly upregulated and expressed in an average of 77% of PAX7-expressing MuSCs on a single EDL myofiber (Figures 3B and 3C). We confirmed that 98% of tdTomato (+) cells associated with EDL myofibers after 24 h of culture were MYOD (+) (Figures 3D and 3E). During the *ex vivo* culture of MuSCs associated with single EDL myofibers for 48 h MuSCs expanded and activated the late myogenic differentiation program by expressing myogenin (MYOG). We confirmed the colocalization of endogenous MYOD with tdTomato in proliferating MuSCs on a single EDL myofiber after 48 h in culture (Figure S6A), and a fraction of tdTomato (+) MuSCs also expressed MYOG (Figure S6B). To further assess the fidelity of the tdTomato fluorescence in MuSCs of *MyoD*^*KI/+*^ mice*,* we cultured MACS-isolated MuSCs for 4 days and verified that an average of 98% of tdTomato (+) cells were positive for MYOD (+) (Figures 3F and 3G). To analyze the tdTomato fluorescence during myogenic differentiation *in vitro,* we isolated MuSCs from adult *MyoD*^*KI/+*^ mice and cultured them in a differentiation medium (DM) for either 3 or 8 days. Immunofluorescence analysis of MYOD and tdTomato shows that the tdTomato-expressing myotubes accumulate MYOD in the myonuclei at day 3 (Figure S5B). However, by day 8, while the accumulation of MYOD protein in the myonuclei became fuzzy, the tdTomato signal weakly remained in those myotubes as depicted by the yellow box in Figure S5C.

Activated MuSCs are heterogeneous populations that associate with distinct myogenic states. On day 4 in culture, a majority of MuSCs expressing MYOD undergo proliferation or differentiation, while a small number show MYOD downregulation, thereby maintaining an undifferentiated state representing “reserve cell” population.^11^^,^^26^^,^^28^ We observed weak or no MYOD-expressing MuSCs from *MyoD*^*KI/+*^ mice (Figure 3F). Notably, cells with low MYOD expression appeared to have low tdTomato expression (Figure 3F, white arrows). Further, flow cytometry analysis demonstrated that cultured MuSCs expressing low and high levels of tdTomato were distinguishable (Figure 3H). To verify that tdTomato fluorescence intensity can recapitulate endogenous MYOD protein expression level, MuSCs expressing tdTomato at low and high levels were isolated using flow cytometer and immunostained for MYOD and tdTomato (Figure 3I). MuSCs expressing low tdTomato fluorescence exhibited low endogenous MYOD protein levels and vice versa (Figures 3J and 3K). Finally, we found a positive correlation between tdTomato fluorescence and MYOD protein levels (*R*^*2*^*=0.4154,* p *< 0.0001*) when each cell was plotted (Figure 3L). Taken together, the *MyoD*^*KI/+*^ reporter mouse is a useful tool for monitoring endogenous MYOD dynamics *in vitro*, particularly during the early activation phase of myogenesis.

### Homozygous MyoD-KI mice (*MyoD*^*KI/KI*^) show no abnormality in growth and myogenic differentiation

**Figure 4:**
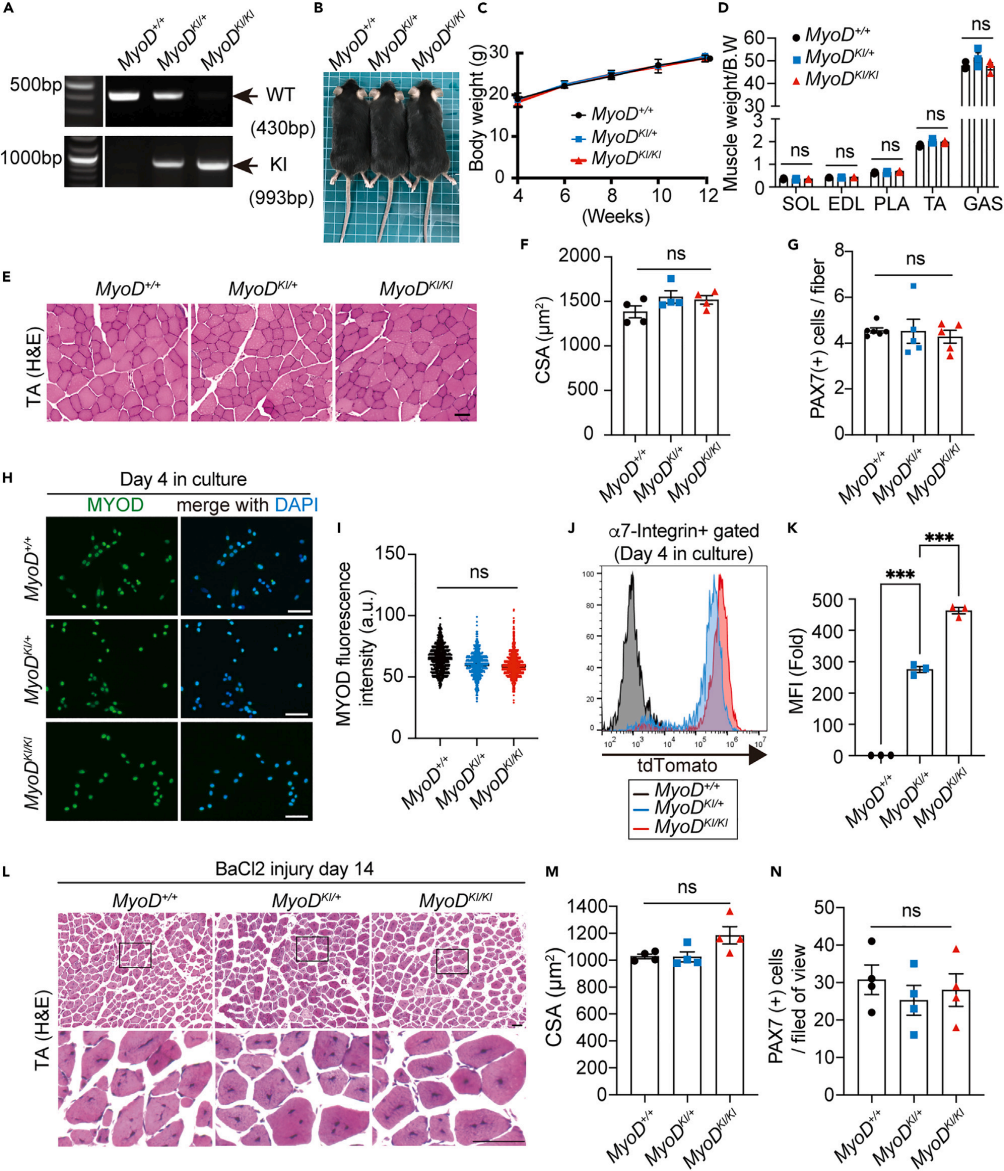
Endogenous MYOD expression is unchanged in *WT*, *MyoD*^*KI/+*^, and *MyoD*^*KI/KI*^ mice (A) Genomic DNA analysis from tail clips of *MyoD*^*+/+*^*(WT)*, *MyoD*^*KI/+*^ (hetero), or *MyoD*^*KI/KI*^ (homo) mice using PCR. The size of PCR products amplified from the WT allele (430 bp) and KI allele (993 bp) is indicated. (B) Representative image of *MyoD*^*+/+*^, *MyoD*^*KI/+*^, and *MyoD*^*KI/KI*^ mice at 12 weeks of age. (C) Body weight changes in male *MyoD*^*+/+*^, *MyoD*^*KI/+*^, and *MyoD*^*KI/KI*^ mice at 4–12 weeks of age (*MyoD*^*+/+*^, n = 5; *MyoD*^*KI/+*^, n = 7; *MyoD*^*KI/KI*^, n = 7). (D) Skeletal muscle weights of 12-weeks-old male *MyoD*^*+/+*^, *MyoD*^*KI/+*^, and *MyoD*^*KI/KI*^ mice, normalized to body weight (n = 3 mice/genotype). (E) Hematoxylin and eosin staining of TA muscle cross-sections. Scale bar: 50 μm. (F) Cross-sectional areas of TA muscles of male *MyoD*^*+/+*^, *MyoD*^*KI/+*^, and *MyoD*^*KI/KI*^ mice at 12 weeks of age (n = 4 mice/genotype). (G) Average number of PAX7 (+) cells per EDL myofiber of male *MyoD*^*+/+*^, *MyoD*^*KI/+*^, and *MyoD*^*KI/KI*^ mice at 12–16 weeks of age (*MyoD*^*+/+*^, n = 6; *MyoD*^*KI/+*^, n = 5; *MyoD*^*KI/KI*^, n = 5). (H) Immunofluorescence staining of MYOD (green) after 4-day culture of MACS-isolated MuSCs from 15 to 16-week-old male *MyoD*^*+/+*^, *MyoD*^*KI/*+^ or *MyoD*^*KI/KI*^ mice (n = 3 mice/genotype). Nuclei were stained with DAPI. Scale bar: 50 μm. (I) Quantification of MYOD fluorescence intensity in H (*MyoD*^*+/+*^, n = 982; *MyoD*^*KI/+*^, n = 690; *MyoD*^*KI/KI*^, n = 1041). (J) Representative flow cytometry histogram of cultured MuSCs isolated from *MyoD*^*+/+*^, *MyoD*^*KI/+*^, and *MyoD*^*KI/KI*^ mice. (K) Quantification of MFI of tdTomato in J (n = 3 mice/genotype). (L) Hematoxylin and eosin staining of TA muscle cross-sections from male *MyoD*^*+/+*^, *MyoD*^*KI/+*^, and *MyoD*^*KI/KI*^ mice at 13–15-week of age, 14 days post BaCl_2_ injury. The area in white dotted boxes is shown at higher magnification. Scale bar: 50 μm. (M) Cross-sectional areas of TA muscles of male *MyoD*^*+/+*^, *MyoD*^*KI/+*^, and *MyoD*^*KI/KI*^ mice at 13–15-week of age, 14 days post BaCl_2_ injury. (n = 4 mice/genotype). (N) The numbers of PAX7 (+) cells on cross-sections of TA muscles from *MyoD*^*+/+*^, *MyoD*^*KI/+*^, and *MyoD*^*KI/KI*^ mice at 13–15-week of age, 14 days post BaCl_2_ injury. (n = 4 mice/genotype). All data are represented as the mean ± s.e.m. ∗∗∗p < 0.001, ns, not significant.

Previously, MyoD-null MuSCs exhibited increased basal numbers, delayed entry into the cell cycle, and defects in proliferation and differentiation. Therefore, we investigated whether function was maintained in MyoD-KI homozygous mice (*MyoD*^*KI/KI*^) (Figure 4A). Both *MyoD*^*KI/KI*^ and *MyoD*^*KI/+*^ mice grew normally, similar to the wild type (WT, *MyoD*^*+/+*^) mice with respect to body and muscle tissue weight (Figures 4B–4D). No abnormal structure of myofibers with central nucleation or reduction in myofiber cross-sectional area was observed in mice as assessed using hematoxylin and eosin staining (Figures 4E and 4F). Furthermore, the number of MuSCs associated with single EDL myofibers remained unchanged in *MyoD*^*+/+*^, *MyoD*^*KI/+*^, and *MyoD*^*KI/KI*^ mice (Figure 4G). Subsequently, comparison of the endogenous MYOD expression levels in cultured MuSCs from all three genotypes using immunofluorescence indicated similar levels of MYOD expression (Figures 4H and 4I). Conversely, tdTomato fluorescence intensity was higher in *MyoD*^*KI/KI*^ mice than that in *MyoD*^*KI/+*^ mice, suggesting that the MYOD protein was translated from transcripts originating from both alleles (Figures 4J and 4K).

Next, we analyzed the regenerative ability in *MyoD*^*+/+*^*, MyoD*^*KI/+*^*,* and *MyoD*^*KI/KI*^ mice. We injected BaCl_2_ into TA muscles of *MyoD*^*+/+*^, *MyoD*^*KI/+*^, and *MyoD*^*KI/KI*^ mice. At day 14 after injury, CSA and the number of PAX7 (+) cells were examined. Our results show that both CSA and the number of PAX7 (+) were almost identical among the three genotypes, suggesting that the insertion of tdTomato at the *MyoD* locus does not impair MuSCs function even under strong regenerative stress (Figures 4L–4N). Together, these data demonstrated that tdTomato KI at the *MyoD* locus does not inhibit endogenous MYOD expression and muscle development and regeneration.

### MyoD-tdTomato fluorescence intensity defines the activation status of MuSCs

**Figure 5:**
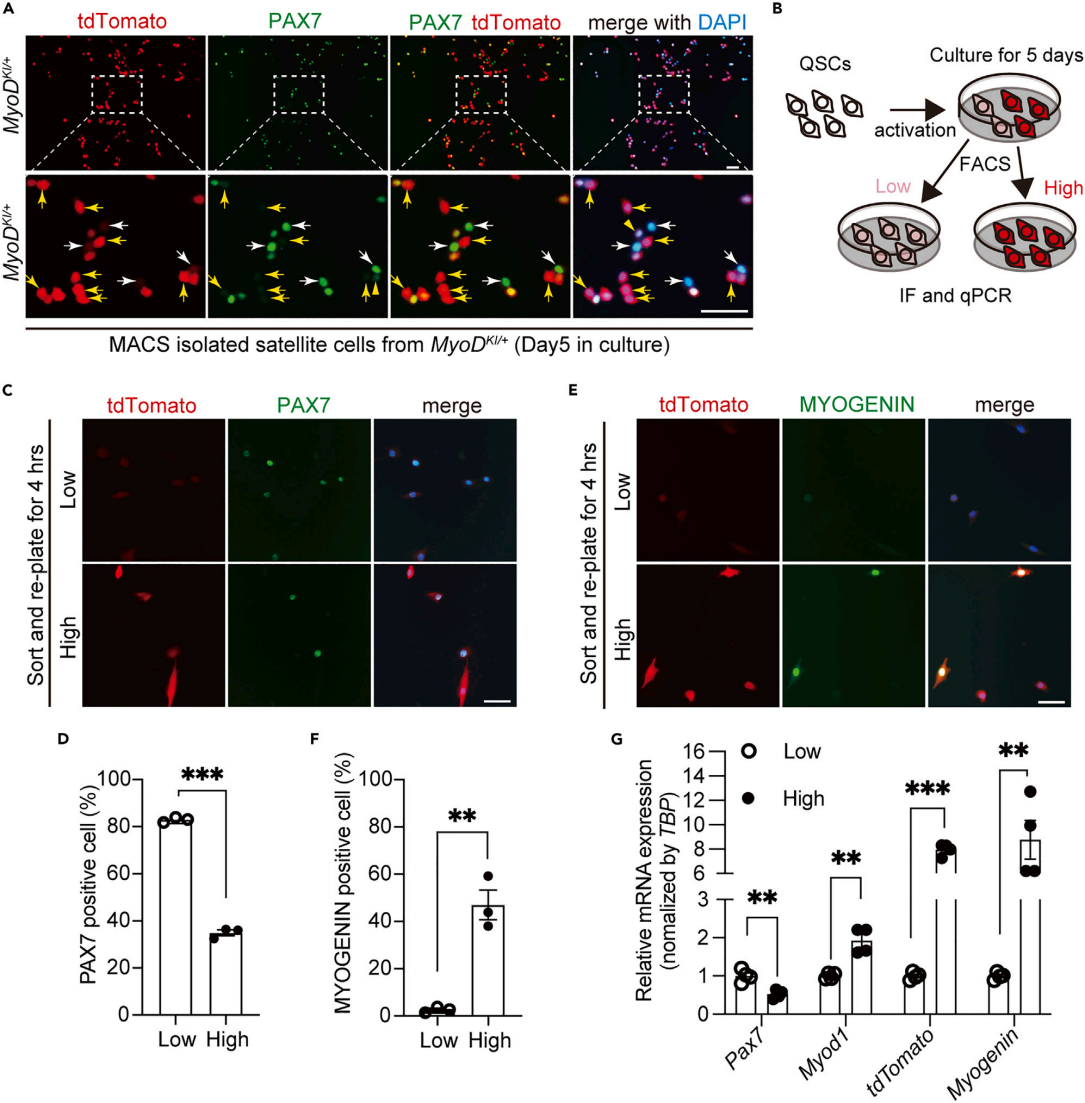
MyoD-tdTomato fluorescence patterns reflect the dynamic status of MuSCs (A) Immunofluorescence of PAX7 (green) and tdTomato (red) on cultured MuSCs from 15 to 16-week-old *MyoD*^*KI/+*^ mice. The area in white dotted boxes is shown at a higher magnification. White arrows indicate that the MuSCs expressing tdTomato weakly have a strong PAX7 expression. Yellow arrows indicate that the MuSCs expressing tdTomato strongly have undetectable PAX7 expression. Scale bar: 50 μm. (B) Schematic diagram of MyoD-tdTomato^low^ and MyoD-tdTomato^high^ populations *in vitro*. (C–F) Cultured MuSCs isolated from *MyoD*^*KI/+*^ mice are sorted using flow cytometer into MyoD-tdTomato^low^ and MyoD-tdTomato^high^ populations, and these cells are re-plated for 4 h to perform immunofluorescence analysis PAX7 (C) or MYOG (E) expression with tdTomato. Scale bar: 50 μm. (D, F) The proportion of PAX7 (+) or MYOG (+) nuclei in DAPI (+). (n = 3 independent experiments) (G) Relative *Pax7, MyoD*, *tdTomato,* and *Myogenin* expression levels, determined by RT-qPCR, in MyoD-tdTomato^low^ and MyoD-tdTomato^high^ populations (n = 4 independent experiments). *TBP, TATA-box binding protein.* All data are represented as the mean ± s.e.m. ∗∗p < 0.01, ∗∗∗p < 0.001.

*MyoD*^*KI/+*^ reporter mice recapitulated MYOD expression without interrupting its endogenous function; therefore, we investigated whether tdTomato fluorescence defines the state of MuSCs during myogenesis *in vitro*. First, MACS-isolated MuSCs were cultured for five days and assessed using immunofluorescence for PAX7 and tdTomato expression (Figure 5A). Heterogeneous tdTomato fluorescence patterns were observed in MuSCs at this stage. Of note, MuSCs expressing low levels of tdTomato expressed high levels of PAX7 (Figure 5A, white arrows) and vice versa (Figure 5A, yellow arrows). To confirm that tdTomato fluorescence intensity correlates with the myogenic state of MuSCs, cultured MuSCs expressing low and high tdTomato (MyoD-tdTomato^low^ and MyoD-tdTomato^high^) were sorted using flow cytometry (Figure 5B). Immunofluorescence labeling for PAX7 and tdTomato showed that around 80% of MuSCs in the MyoD-tdTomato^low^ were positive for PAX7. This rate was significantly reduced to an average of 35% in the MyoD-tdTomato^high^ (Figures 5C and 5D). We simultaneously performed immunofluorescence analysis for MYOG. MYOG-expressing MuSCs in the MyoD-tdTomato^low^ population were almost undetectable (Figures 5E and 5F). Conversely, an average of 47% of MuSCs in the MyoD-tdTomato^high^ population expressed MyoG (Figures 5E and 5F). Additionally, the distinct characteristics between the two populations were further confirmed using real-time quantitative PCR (RT-qPCR) for *Pax7*, *MyoD, tdTomato,* and *Myogenin* (Figure 5G), thereby indicating that the MyoD-tdTomato^low^ population was enriched with undifferentiated stem-like cells, and the MyoD-tdTomato^high^ population contained committed proliferating and MYOG (+) cells.

### RNA-seq analysis of the MyoD-tdTomato^low^ and MyoD-tdTomato^high^ populations revealed unique gene expression profiles

**Figure 6:**
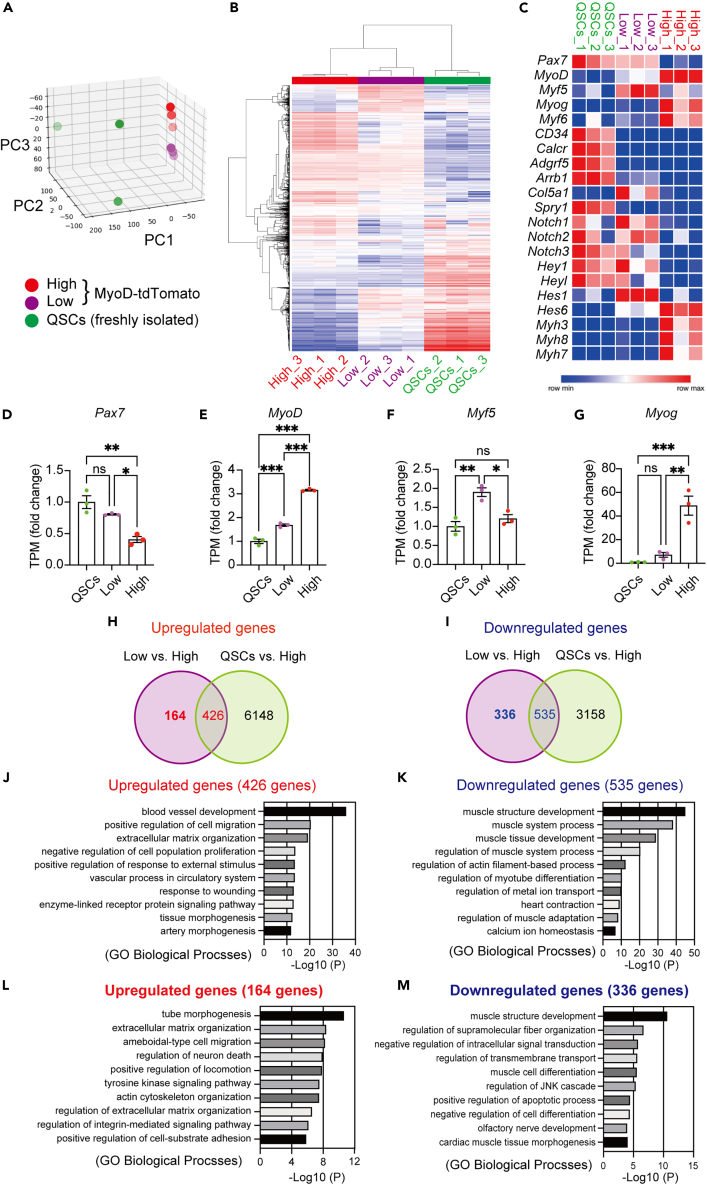
Global gene expression analysis of MyoD-tdTomato^low^ and MyoD-tdTomato^high^ populations shows distinct gene expression profiles (A) The 3D principal component analysis (PCA) plots of gene expression in freshly isolated MuSCs from Pax7-YFP mice (QSCs), FACS-sorted MyoD-tdTomato^low^ and MyoD-tdTomato^high^ population after 5 days culture. (n = 3 per group). (B) Heatmap displaying differential gene expression in QSCs, MyoD-tdTomato^low^, and MyoD-tdTomato^high^ (n = 3 per group). (C) Heatmap showing expression of MRFs, muscle stem cell quiescence- and activation-related genes (n = 3 per group). (D–G) Transcripts per million levels of *Pax7*, *MyoD*, *Myf5*, and *Myog* analyzed by RNA-seq in the QSCs, MyoD-tdTomato^low^, and MyoD-tdTomato^high^ (n = 3 per group). (H) Venn diagram showing the genes significantly upregulated in MyoD-tdTomato^low^ or QSCs compared with MyoD-tdTomato^high^ (FDR < 0.01, fold change > 1.5). (I) Venn diagram showing the genes significantly downregulated in MyoD-tdTomato^low^ or QSCs when compared with MyoD-tdTomato^high^ (FDR < 0.01, fold change > 1.5). (J–K) Gene ontology enrichment analysis of commonly upregulated (J) and downregulated (K) genes in MyoD-tdTomato^low^ and QSCs compared to MyoD-tdTomato^high^. (L and M) Gene ontology enrichment analysis of specifically upregulated (L) and downregulated (M) in MyoD-tdTomato^low^ compared to MyoD-tdTomato^high^. The top 10 enriched gene sets are shown. All data are represented as the mean ± s.e.m. ∗p < 0.05, ∗∗p < 0.01, ∗∗∗p < 0.001, ns, not significant.

*MyoD*^*KI/+*^ mice can distinguish undifferentiated (MyoD-tdTomato^low^) and committed (MyoD-tdTomato^high^) MuSCs from heterogeneous populations in culture, which has been challenging to explore this far. Therefore, obtaining whole-genome expression profiles from these two populations would greatly benefit the identification of new regulatory genes in MuSCs. We performed RNA sequencing of these two populations isolated using FACS after 5-day of culture. Further, we analyzed the whole gene expression in freshly isolated MuSCs from Pax7-YFP KI mice.^32^ Principal component analysis revealed a clear sample separation among groups (Figure 6A). As expected, the transcriptome profile of freshly isolated MuSCs (QSCs) was distinct from that of MyoD-tdTomato^low^ and MyoD-tdTomato^high^ populations (Figure 6A). We detected 1479 genes that were differentially expressed between the MyoD-tdTomato^low^ and MyoD-tdTomato^high^ populations, visualized using a heatmap (Figure 6B). Additionally, we visualized selected genes related to MuSC quiescence (*Pax7, CD34, Calcr, Col5a1, Spry1, Adgrf5, Arrb1, Notch1-3, Hey1,* and *Heyl*), activation (*MyoD, Myf5,* and *Hes1*), and differentiation (*Myog, Myf6, Hes6, Myh3, Myh7, and Myh8)* among the groups (Figure 6C). The quiescence marker genes were abundantly expressed in QSCs, while some genes overlapped with the MyoD-tdTomato^low^ population. When we compared the quiescence marker genes expression level between the QSCs and MyoD-tdTomato^Low^ populations, these markers were significantly higher in QSCs than the MyoD-tdTomato^low^ population, except for Pax7 and Myf5 (Figures 6C, 6D, 6F). The activation and differentiation genes were more accumulated in the MyoD-tdTomato^high^ population than the MyoD-tdTomato^low^ population (Figures 6C–6G). Together, whole gene expression profiles also support the notions that MyoD-tdTomato^low^ population represent undifferentiated stem-like cells with intermediate characteristics between quiescent and committed/activated MuSCs, and the MyoD-tdTomato^high^ population represents committed proliferating and differentiating cells.

To further characterize MyoD-tdTomato^low^ and MyoD-tdTomato^high^ populations, we performed gene ontology (GO) analysis on differentially expressed genes (DEGs). Comparison of MyoD-tdTomato^low^ with MyoD-tdTomato^high^ revealed 590 upregulated and 871 downregulated genes in the MyoD-tdTomato^low^ population. Of the 590 upregulated genes, 426 genes were commonly upregulated between MyoD-tdTomato^low^ vs. MyoD-tdTomato^high^ and QSCs vs. MyoD-tdTomato^high^ (Figure 6H). Of the 871 downregulated genes, 535 genes were commonly downregulated between MyoD-tdTomato^low^ vs. MyoD-tdTomato^high^ and QSCs vs. MyoD-tdTomato^high^ (Figure 6I). Among the 426 upregulated genes, there was a strong enrichment for terms related to blood vessel development, extracellular matrix (ECM) organization, and tissue morphogenesis (Figure 6J, Tables S1 and S2). In contrast, among the 535 downregulated genes, there was a strong enrichment for terms related to muscle structure/tissue development and myotube differentiation (Figure 6K, Tables S1 and S2).

Since our *MyoD*^*KI/+*^ model allowed us to obtain a unique intermediate stage of MuSCs (Myod-tdTomato^low^) wherein they express high levels of *Pax7* and *Myf5,* and low *MyoD in vitro,* next, we considered the genes that were exclusively upregulated or downregulated in the MyoD-tdTomato^low^ population without corresponding changes reported in QSCs vs. MyoD-tdTomato^high^ (Figures 6H-6I). Interestingly, within 164 genes upregulated in Myod-tdTomato^low^ population, those associated with tube morphogenesis, including *Angpt1*, *Foxc2*, *Cxcl12*, *Csf1*, *Mmp14*, and *Smad7* (Figure 6L, Tables S1 and S2) was determined to be the most significantly represented class of genes, consistent with previous observations.^41^^,^^42^^,^^43^^,^^44^^,^^45^ The second most significant class of genes was those involved in ECM organization, which includes *Col4a2, Has2*, *Fn1*, *lamb1*, and *Nid1* (Figure 6L, Tables S1 and S2).^46^^,^^47^^,^^48^^,^^49^ In contrast, among the 336 genes downregulated exclusively in Myod-tdTomato^low^ population, there was a strong enrichment for terms related to muscle structure development and muscle cell differentiation (Figure 6M, Tables S1 and S2). These transcriptome data of cultured MuSCs from our *MyoD*^*KI/+*^ mice will be a unique resource for elucidating the undefined molecules associated with stemness properties in MuSCs for future research.

### MyoD-KI mice can be used to identify new molecular targets for the quiescence and self-renewal of MuSCs *in vitro*

**Figure 7:**
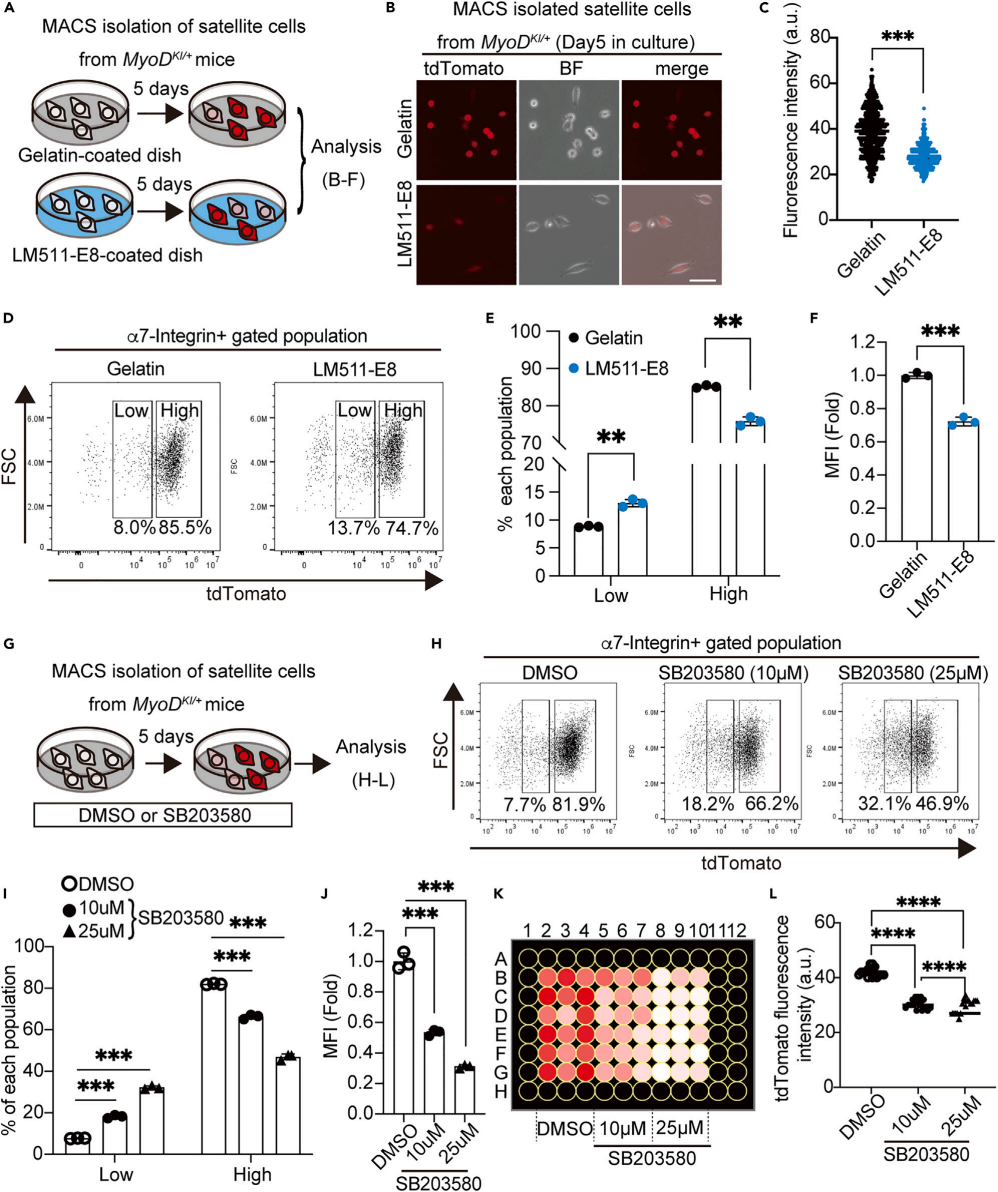
*MyoD*^*KI/+*^ mice are useful for drug screening to manipulate MuSCs *in vitro* (A) Schematic diagram of culture of MuSCs from *MyoD*^*KI/+*^ mice in gelatin- and LM-511-E8-coated dishes. (B) Representative fluorescence images of MuSCs isolated from *MyoD*^*KI/+*^ mice and cultured for 5 days in either gelatin- or LM511-E8-coated dishes. Scale bar: 50 μm. (C) Quantification of MyoD-tdTomato fluorescence intensity in MuSCs in (B) (Gelatin, n = 717; LM511-E8, n = 446). (D) Representative flow cytometry plot of cultured MuSCs isolated from *MyoD*^*KI/+*^ mice in either gelatin- or LM511-E8-coated dishes. (E and F) Quantification of MyoD-tdTomato^Low^ and MyoD-tdTomato^high^ fractions (E) and MFI (F) in (D). The gate is defined based on the WT-derived MuSCs analysis. (n = 3 mice per group). (G) Schematic diagram of culture of MuSCs from *MyoD*^*KI/+*^ mice in the presence of DMSO or SB203580. (H) Representative flow cytometry plot of MuSCs isolated from *MyoD*^*KI/+*^ mice and cultured for 5 days with 10, 25 μM of SB203580, or DMSO control. (I–K) Quantification of MyoD-tdTomato^Low^ and MyoD-tdTomato^high^ fractions (I) and MFI (J) in H (n = 3 mice per group). (K) MuSCs from *MyoD*^*KI/+*^ mice are cultured in a 96-well-plate for 5 days with 10, 25 μM of SB203580, or DMSO control. MyoD-tdTomato fluorescence intensity is visualized as heatmap. (L) MyoD-tdTomato fluorescence intensity of each well in K is quantified. All data are represented as the mean ± s.e.m. ∗∗p < 0.01, ∗∗∗p < 0.001.

Our data suggest that the MyoD-tdTomato^low^ population displays an undifferentiated state and expresses defined stem cell-like markers, whereas the MyoD-tdTomato^high^ population exhibits committed/proliferative myogenic progenitor features (Figures 5 and 6). Therefore, we hypothesized that monitoring the fluorescence pattern/ratio of MyoD-tdTomato would enable us to screen the global activation state of cultured MuSCs, which has been difficult to analyze so far without immunostaining. The laminin 511-E8 fragment (LM511-E8) is an important ECM component in the maintenance of stemness in MuSCs.^50^ Thus, we cultured MACS-isolated MuSCs on either gelatin or LM511-E8 for 5 days (Figure 7A), and tdTomato fluorescence intensity and ratio in the MuSCs were analyzed by microscopically (Figures 7B and 7C) or by flow cytometry (Figures 7D–7F). MuSCs cultured on LM511-E8 exhibited low levels of tdTomato fluorescence and a higher percentage of MyoD-tdTomato^low^ population than those cultured on gelatin (Figures 7B–7F).

In addition to the substrate for MuSC culture, we tested whether the *MyoD*^*KI/+*^ mice would be a valuable tool for assessing the effect of compounds on MuSC status in a high-throughput manner. We cultured MACS-isolated MuSCs in the presence of SB203580, a p38 MAPK inhibitor that is an established target for maintaining stemness in MuSCs (Figure 7G).^29^^,^^30^^,^^51^ FACS analysis of the MuSCs demonstrated that the p38 MAPK inhibitor significantly maintained the MyoD-tdTomato low population in a dose-dependent manner, when compared with the dimethyl sulfoxide (DMSO) control (Figures 7H–7J), thereby indicating that the *MyoD*^*KI/+*^ mouse line is useful for screening a potential compound/substrate on the effect of MuSC fate decision.

Furthermore, to develop a more efficient screening platform for validating the effect of drugs and substrates on the effects of the myogenic state using a *MyoD*^*KI/+*^ model, we established a 96-well-based fluorescence image quantification screening system using an image cytometer equipped with KEYENCE’s microscope. After 5-day of MuSCs culture with DMSO or SB203580 in a 96-well-plate, the tdTomato fluorescence intensity of each well was captured, and the data were visualized using a heatmap (Figure 7K) and a plot graph (Figure 7L). Consistent with the results of the FACS analysis, we detected that SB203580 significantly suppressed MyoD-tdTomato fluorescence in a dose-dependent manner when compared to the DMSO control (Figures 7K and 7L). Therefore, these data suggest that this screening system using *MyoD*^*KI/+*^ mice will be beneficial for identifying new substances that regulate the fate of MuSCs *in vitro.*

## Discussion

Herein, using CRISPR-Cas9, we generated a KI mouse line that recapitulates endogenous MYOD expression using a tdTomato fluorescent reporter *in vivo* and *in vitro*. Paradoxically, *MyoD* transcripts are present but absent at the protein level in quiescent MuSCs, which is controlled by translational suppression by RBPs, such as Stau1, through the M*yoD* 3′-UTR.^15^ Therefore, we first evaluated whether CRISPR-Cas9-mediated gene editing on MyoD 3′-UTR with the addition of the tdTomato sequence would not disrupt the translational suppression of *MyoD in vitro*. If the addition of a tdTomato sequence changes the accessibility of Stau1 to the MyoD 3′-UTR, aberrant translation of MYOD is induced. We confirmed that Stau1-mediated translational suppression is preserved in the tdTomato-MyoD 3′-UTR sequence. We successfully obtained the F0 founder and established the MyoD-KI mouse line, with no inappropriate mutations in the endogenous MyoD sequence, including the stop codon (Figures S8 and S9). We confirmed the restricted expression of MyoD-tdTomato in myogenic progenitors and activated MuSCs but not in quiescent MuSCs. Additionally, homozygous *MyoD*^*KI/KI*^ mice are viable with no morphological abnormalities in skeletal muscle and expression levels similar to endogenous MYOD in *WT* or *MyoD*^*KI/+*^ mice.

Importantly, we found that MyoD-tdTomato fluorescence intensity mirrors the endogenous MYOD protein expression in cultured MuSCs. While it is clear that not all quiescent MuSCs are functionally homogeneous,^38^ their progeny are diverse, especially when activated. Previous studies using a label-retaining assay have revealed distinct populations in MuSCs.^34^^,^^35^ MuSCs with limited proliferative output are enriched for self-renewal, whereas fast-dividing MuSCs proceed to differentiation. However, though MYOD is most frequently used to quantify heterogeneity in the MuSC population, no direct evidence of heterogeneity based on MYOD expression has been reported due to the lack of tools to distinguish between MYOD-labeled and -unlabeled MuSCs. To the best of our knowledge, our study is the first to isolate and analyze heterogeneous MuSC populations based on MYOD expression.

We performed RNA-seq analysis to compare whole transcriptome expression profiles in MyoD-tdTomato low- and high-expressing MuSCs and freshly isolated MuSCs. RNA-seq and protein analysis confirmed that the MyoD-tdTomato^low^ population was enriched in undifferentiated MuSC features, and the MyoD-tdTomato^high^ population showed committed and differentiating cell features. Previously reported quiescent MuSC-associated genes, such as *CD34*, *Calcr, Spry1*, *and Notch1–3*, are highly expressed in freshly isolated MuSCs. *Adgrf5*, an adhesion G protein-coupled receptor 116, and its downstream effecter *Arrb1*, important for the quiescence and long-term maintenance of the MuSC pool, as we previously reported,^52^ are also enriched in this population. Notably, the expression of these quiescence signatures is downregulated in the MyoD-tdTomato^low^ population but remains highly maintained when compared to the MyoD-tdTomato^high^ population. Intriguingly, *Pax7* expression was not significantly different between freshly isolated MuSCs and MyoD-tdTomato^low^ population. Furthermore, we found that the *Myf5* transcript was highly enriched in the MyoD-tdTomato^low^ population when compared to the freshly isolated MuSCs and the MyoD-tdTomato^high^ population. *Myf5* and *MyoD* functionally compensate for each other in myogenic lineage determination during embryonic development but play distinct roles in adult regeneration.^22^^,^^25^^,^^53^ Moreover, the absence of *MyoD* leads to upregulation of *Myf5* and an increase in the number of MuSCs as a consequence of increased self-renewal rather than myogenic differentiation.^22^^,^^25^ Therefore, we interpret these data to suggest that the MyoD-tdTomato^low^ population expressing high levels of *Pax7* and *Myf5* contains a rare intermediate state between quiescent and activated MuSCs.^23^^,^^25^^,^^54^

Our whole transcriptome data will be a great database to decipher the new molecular mechanism for a small unique subset of self-renewing MuSCs and to determine their fate in future studies. We found that the MyoD-tdTomato^low^ population expresses genes associated with endothelial cell development. Interestingly, the endothelial and skeletal muscle lineages in the trunk and limbs arise from common embryonic progenitors.^42^^,^^55^ Furthermore, the angiopoietin 1/Tie-2 axis, which is highly enriched in the MyoD-tdTomato^low^ population, regulates self-renewal of MuSCs.^41^ It is tempting to hypothesize that genes enriched in the MyoD-tdTomato^low^ population recapitulate, at least in part, myogenesis by common multipotent progenitors. Using our database as a stepping stone, the functions of these undefined genes can be verified to unveil the new paradigm of MuSC research.

MYOD expression has been used as an activation marker for MuSCs for several decades. However, there is a lack of tools to efficiently quantify MYOD (+) or (−) cells. Usually, the fraction of immunolabeled cells positive for PAX7 and/or MYOD is quantified to indicate self-renewed, activated, and committed MuSCs in cultured MuSCs or on the muscle cross-sections. However, such visual assessment of images by individuals can lead to bias in their perception of patterns in the images and in subsequent interpretation of data. Additionally, the activated MuSCs also exhibit heterogeneous MYOD expression patterns, with some showing bright and others showing weak MYOD; thus, the visual evaluation of immunolabeled cells by individuals is limited in reproducibility. Here, we propose a model in which MyoD-tdTomato fluorescent pattern/intensity efficiently identifies undifferentiated stem-like or proliferative/committed MuSCs using a non-biased approach, such as flow cytometry, without immunolabeling. Therefore, this unbiased method using MyoD-KI mice is expected to contribute to the replicability and reproducibility in skeletal muscle research.

Furthermore, a high-throughput system for comparing the effects of compounds in regulating MuSC self-renewal and expansion has not yet been established, although many studies have attempted to expand MuSCs without losing stem cell regenerative capacity.^13^^,^^30^^,^^31^^,^^50^ We developed a platform to screen compounds for manipulating MuSC fates based on monitoring MyoD-tdTomato fluorescence intensity using an image cytometer. Since MuSCs are rare, a 96-well-plate system could be a beneficial screening system for validating many candidates from the library for their *ex vivo* expansion toward regenerative medicine.

In summary, we successfully developed a MyoD-KI mouse line to monitor MYOD expression using a tdTomato fluorescent reporter, without interfering with endogenous MYOD protein expression during myogenesis. Furthermore, MyoD-tdTomato fluorescence intensity partially recapitulates the myogenic progression of MuSCs *in vitro* and *in vivo.* Therefore, our MyoD-KI mice can be used as a screening tool to easily and efficiently distinguish between undifferentiated and committed MuSCs under various conditions, including knockout of a gene of interest or in the presence of an undefined compound of interest. Moreover, our MyoD-KI mice and the RNA-seq database in the present study will be valuable resources for identifying a key molecule regulating a small subset of the reserve MuSC population during myogenesis.

### Limitations of the study

Our study reveals that the tdTomato sequence immediately before the *MyoD 3′UTR* sequence does not interfere the post-translational regulation by Stafune1 in HEK293T cells, but does not investigate the context of myogenic progenitors. Although, we confirmed that the adult MuSCs from *MyoD*^*KI/+*^ mice specifically express tdTomato upon activation, further investigation is required to reveal whether the post-translational regulators of *MyoD 3′UTR*, such as Staufen1^15^ and Tristetraprolin (TPP),^14^ can be functional in the MuSCs from *MyoD*^*KI/+*^ mice.

We have shown that MyoD-tdTomato fluorescence successfully recapitulates the endogenous MYOD protein levels under several conditions. However, we must point out that MyoD-tdTomato fluorescence is not perfect in some conditions as MyoD expression dynamically changes during myogenesis. For instance, at day 14 after BaCl_2_ injury, only 49% of tdTomato signals are successfully overlapped with endogenous MYOD signals detected by the antibody. This could be attributed to the fact that the tdTomato protein has a longer half-life than MYOD in MuSCs, wherein MyoD expression has already been downregulated or tuned off. Future research using these *MyoD*^*KI/+*^ mice should consider this limitation.

Moreover, our current evidence supports that tdTomato signals in the cryosections of E10.5, E13.5 embryos, and injured TA muscles, are primarily restricted to the dermomyotome area, muscle-forming regions, and regenerating myofibers, respectively. However, it remains unclear whether the tdTomato signals detected in the cryosections of E10.5 and E13.5 embryos, and in the regenerating myofibers, are positive for endogenous MYOD. This is because the MYOD antibody used in the present study has limitations in detecting MYOD on the PFA-prefixed mouse cross-sections in our protocol.

We also note that the molecular features of the MyoD-tdTomato-negative population remain unknown. We purified quiescent MuSCs from *MyoD*^*KI/+*^ mice by using MACS and culture expansion. However, MACS-based isolation showed a slight contamination of non-myogenic cells, which are completely negative populations for MyoD-tdTomato (Figures S7A–S7C). Thus, we were unable to perform whole transcriptome analysis of the MyoD-tdTomato-negative population in the present study. Nonetheless, there would be a very minor MyoD-tdTomato negative MuSC population, which may represent a rare subset of stress-resistant MuSCs expressing Pax3, as reported previously.^6^^,^^8^ In the future, the combination of our MyoD-KI mice with single-cell RNA-seq will help identify the population and its molecular signatures. This experiment is currently underway in our laboratory.

## STAR★Methods

### Key resources table

**Table:** 

REAGENT or RESOURCE	SOURCE	IDENTIFIER
**Antibodies**		

anti-PAX7	Santa Cruz	Cat# sc-81648; RRID: AB_2159836
anti-MYOD	Santa Cruz	Cat# sc-377460, RRID: AB_2813894
anti-MYOGENIN	abcam	Cat# ab124800, RRID: AB_10971849
anti-RFP	Rockland	Cat# 600-401-379, RRID: AB_2209751
Alexa Fluor 488 goat anti-mouse IgG1	Thermo Fisher Scientific	Cat# A-21121, RRID: AB_2535764
Alexa Fluor 488 goat anti-mouse IgG	Thermo Fisher Scientific	Cat# A-11001, RRID: AB_2534069
Alexa Fluor 546 goat anti-rabbit IgG	Thermo Fisher Scientific	Cat# A-11010, RRID: AB_2534077
anti-mouse integrin α-7 MicroBeads	Miltenyi Biotec	Cat# 130-104-261

**Chemicals, peptides, and recombinant proteins**		

DMEM	Thermo Fisher Scientific	Cat# 11965-092
F12	Thermo Fisher Scientific	Cat# 11765-054
Collagenase D	Roche	Cat# 11088866001
D-PBS (−)	FUJIFILM	Cat# 045-29795
OPTI-MEM	Thermo Fisher Scientific	Cat# 31985062
iMatrix-511	nippi	Cat# 892 012
Histomount	national diagnostics	Cat# HS-103
1X RBC Lysis Buffer	pluriSelect	Cat# 60-00050-13
Proteinase K Solution	KANTO CHEMICAL. CO, INC.	Cat# 34060-97
M.O.M. Blocking Reagent	Vector	Cat# MKB-2213-1
Barium chloride	MERK	Cat# 449644
TRIzol	Thermo Fisher Scientific	Cat# 15596018
Gelatin from porcine skin	MERK	Cat# G1890
Ultroser G	PALL	Cat# 15950-017
Fetal bovine serum	Thermo Fisher Scientific	Cat# 10270-106
Horse serum	Thermo Fisher Scientific	Cat# 26050088
SsoAdvanced™ Universal SYBR Green Supermix	Bio-Rad	Cat# 1725271
4% Paraformaldehyde Fixative	MUTO PURE CHEMICALS CO., LTD.	Cat# 33111
FuGENE 6 Transfection	Promega	Cat# E2692
PrimeSTAR Max DNA	TAKARA BIO Inc.	Cat# R045A
Normal Goat Serum	Vector	Cat# S-1000
VECTASHIELD antifade Mounting Medium	Vector	Cat# H-1000
ISOGEN	NIPPON GENE	Cat# 315-02504

**Critical commercial assays**		

Satellite cell isolation Kit, mouse	Miltenyi Biotec	Cat# 130-104-268
ReverTra Ace™ qPCR RT Master Mix with gDNA Remover	TOYOBO	Cat# FSQ-301
Wizard Plus SV Minipreps DNA Purification System	Promega	Cat# A1460
QIAquick PCR Purification Kit	QIAGEN	Cat# 28104
In-Fusion®HD Cloning Kit	TAKARA BIO Inc.	Cat# Z9649N

**Deposited data**		

Raw RNA-seq data	This paper	GEO:GSE211755

**Experimental models: Cell lines**		

HEK293T	ATCC	SO0623448

**Experimental models: Organisms/strains**		

Mouse: C57BL/6J	The Jackson Laboratory	RRID:IMSR_JAX:000664
Mouse: Pax7-YFP	Kitajima et al.^32^	PMID: 30139390
Mouse:*C57BL/6J-Myod1*^*em1(tdTomato)Utr*^ *(MyoD*^*KI*^*)*	This paper	N/A

**Oligonucleotides**		

See Table S3 for primer sequences	This paper	N/A

**Recombinant DNA**		

px330-mC	RIKEN BRC	RDB14406
pCX-EGFP	Okabe et al.^56^	PMID: 9175875
pCX-tdTomato-MyoD_3′ UTR	This paper	N/A
pCX-Stau1-IRES-EGFP	This paper	N/A

**Software and algorithms**		

BIOREVO BZ-X800 microscope system	Keyence	N/A
Morpheuse	Broad Institute	https://software.broadinstitute.org/morpheus
FlowJo™ v10.8	Becton, Dickinson and Company (BD)	https://www.flowjo.com
Prism	GraphPad	https://www.graphpad.com
Illustrator	Adobe	N/A
Photoshop	Adobe	N/A

**Other**		

MoFlo XDP	BECKMAN COULTER	N/A
7500 Fast Real Time PCR System	Applied Biosystems	N/A
CytoFLEX S	BECKMAN COULTER	N/A
ImageQuant LAS 500	GE Healthcare	N/A

### Resource availability

#### Lead contact

Further information and request for reagents may be directed to and will be fulfilled by the lead contact, Ryo Fujita (fujiryo@md.tsukuba.ac.jp).

#### Materials availability

All unique reagents generated in this study are available from the lead contact in accordance with the relevant material transfer agreements.

### Experimental models and subject details

#### Mice

All experimental procedures were approved by the Animal Experiment Ethics Committee of the University of Tsukuba (authorization number. 190236, 200036). Mice were allowed *ad libitum* access to water and standard rodent chow and were maintained in a pathogen-free facility at the University of Tsukuba. *MyoD*^*KI/+*^ mice were maintained on a C57/BL6 background. The *MyoD*^*KI/+*^ mice generated in this study are named C57BL/6J-Myod1^em1(tdTomato)Utr^, according to the guidelines for the nomenclature of genes, genetic markers, and alleles in mouse and rat (http://www.informatics.jax.org/mgihome/nomen/gene.shtml). Male and female *WT*, *MyoD*^*KI/+*^, *MyoD*^*KI/KI*^ mice, except when indicated, were used in the study. The 15–16-week-old male Pax7-YFP (*Pax7*^YFP/YFP^) mice were used to directly isolate quiescent MuSCs from the leg muscles.^32^ Acute injury of TA muscle was performed by injecting 30 μL of 1.2% BaCl2 (Merk) under anesthesia by isoflurane inhalation.

### Method details

#### Generation of the MyoD-KI construct

We selected a sequence (5′-GCACCTGATAAATCGCATTG-3′) immediately upstream of the MyoD termination codon as the sgRNA. This sequence was inserted into *the px330-mC* plasmid*,* which carries both the guide RNA and Cas9-mC expression units.^57^ There was a P2A-tdTomato sequence between the 5′ AND3' homology arms of the donor DNA *pMyoD-tdTomato*. Genome regions from 1,011 bp upstream to 28 bp upstream of the termination codon in the 5'-homology arm and from the termination codon to 1,100 bp downstream of it in the 3'homology arm were homologous. The isolation of these DNA vectors, microinjection into C57BL/6J mouse zygotes, and embryo transfer were performed as previously described.^57^

#### Construction of pCX-tdTomato-MyoD_3′**-**UTR and pCX-Stau1-IRES-EGFP

The pCX-tdTomato-MyoD_3′-UTR was constructed based on the pCX-EGFP vector.^56^ pCX-EGFP was digested with *Nco*I and *Pst*I to excise the EGFP cDNA and rabbit globin polyA fragments. The tdTomato cDNA and the 3′-UTR of *MyoD* were then introduced into the site where these fragments were originally generated using in-fusion technology. pCX-Stau1-IRES-EGFP was constructed using pCX-EGFP. pCX-EGFP was digested with *EcoR*I to excise EGFP cDNA, and *Stau1* full-length coding sequence (CDS)-IRES-EGFP cDNA was introduced using in-fusion technology. To obtain Stau1 full-length CDS, mRNA was extracted from the liver of C57BL/6J mice using ISOGEN (NIPPON GENE) and reverse transcribed using SuperScript III Reverse Transcriptase (Thermo Scientific, 18080044) and oligo dT primer (Thermo Scientific, SO131). Further, RT-PCR was performed using the following primers: Stau1 forward (Fw) 5′-GCAAAGAATTGCTAGCCACCATGTATAAGCCCGTGGACCCTCACT-3′, Stau1 reverse (Rv) 5′-AGGGAGAGGGGGTTTTCAGCACCTCCCGCACGCTG-3′. Given amount (Figure 1C) of the above DNA vectors was mixed with 200 μL Opti-MEM (Thermo Scientific) and 4 μL of FuGENE 6 transfection reagent (Promega), incubated at room temperature (approximately 23–27°C) for 30 min, and then added to HEK293T cells (ATCC, SO0623448) seeded in 24-well plates. After 24 h, EGFP and tdTomato fluorescence in HEK293T cells was analyzed using a BZ-9000 fluorescence microscope (Keyence) and an appropriate filter OP-87765 or OP-87763 sets (Keyence) or using flow cytometry (CytoFLEX S; Beckman Coulter, Brea, CA, USA).

#### PCR screening of MyoD-KI mice

Genomic DNA was isolated from mouse tail tips using genotyping lysis buffer (100 mM Tris-HCl (pH 8.5), 5 mM EDTA (pH 8.0), 200 mM NaCl, 0.2% SDS) with 100 μg/mL Proteinase K (KANTO CHEMICAL Co., Inc.). The presence of tdTomato in the *MyoD* allele was verified by five screening steps using PCR. First, tdTomato was amplified by PCR (Step 1, Figure 1B). Next, random integration of Cas9 and donor vectors in the mice was confirmed using PCR (Steps 2 and 3). Subsequently, the genomic DNA samples, confirmed in Steps 1–3 were analyzed using the primer pairs indicated in Figure 1B (Step 4, 5). All primers used from step to 5 are listed in Table S3. The full sequence of PCR products amplified in Step 4 and Step 5 was further validated by Sanger sequencing with primers listed in Table S3. We confirmed that the STOP codon and the coding sequence of *MyoD* were appropriately mutated by the insertion of t*dTomato* sequence (Figures S8 and S9). After F2 animals, MyoD-KI heterozygous, homozygous, or wild type mice were distinguished using PCR with the primers listed in Table S3 (Routine genotyping) The expected amplicon sized are 430 and 993 bp for WT and knock-in alleles, respectively (Figure 4A).

#### Muscle stem cell isolation and culture

MuSCs were isolated from the hindlimb muscles of 10–16-week-old mice by magnetic cell sorting (MACS) using a Satellite Cell Isolation Kit (Miltenyi Biotec), together with anti-integrin α7 microbeads (Miltenyi Biotec).^11^^,^^52^ Adult hindlimb muscles were minced in ice-cold Ham’s F12 medium. The minced muscles were then incubated in 0.1% collagenase D (Roche), 0.1% trypsin in Ham’s F12 (Gibco) with 1% penicillin-streptomycin for 45 min at 37 °C on a shaker for three rounds of digestion. After each round, the supernatant was collected in a 50 mL tube containing 8 mL of fetal bovine serum (FBS) on ice, and fresh trypsin/collagenase digestion buffer was added to the tube for the next round of digestion. The collected supernatant was filtered through a sequence of 100 and 40 μm nylon mesh strainers, followed by centrifugation at 400*g* for 10 min at 4 °C. The pellets were resuspended in PBS containing 2% FBS and processed for MACS or flow cytometry. The MuSCs were cultured in a growth medium containing 39% DMEM (Gibco), 39% Ham’s F12, 20% FBS (Gibco), 1% UltroserG (Pall Life Sciences). Differentiation media was 2% horse serum in DMEM. Gelatin (0.2%)-coated dishes were used for MuSC culture, unless otherwise indicated. When indicated, MuSCs were cultured in SB203580 (Selleck) or DMSO (Merck). iMatrix-511-coated dishes were prepared according to the manufacturer’s instructions (nippi).

#### Immunofluorescence

Cultured cells were washed twice with PBS after media removal and fixed using 4% paraformaldehyde (PFA) for 15 min. The cells were permeabilized and blocked using 5% goat serum in 0.1% Triton PBS for 30 min. Single EDL myofibers were fixed using 2% PFA for 15 min, permeabilized with 0.1% Triton in PBS, and blocked with 5% goat serum in 0.1% Triton PBS for 30 min on a shaker. TA muscles were immediately fixed for 2 h in 1% PFA at 4 °C on a shaker and equilibrated overnight in 20% sucrose at 4 °C on a shaker. Further, they were mounted in Tissue Tek O.C.T. compound and frozen in a liquid nitrogen-cooled isopentane bath. Cryosections (10 μm) were subjected to permeabilization using 0.1% triton, 0.1 M glycine in PBS for 5 min and blocked in M.O.M. reagent for 1 h. Immunofluorescence data were obtained using an all-in-one fluorescence microscope BX-X810 (KEYENCE, Osaka, Japan). MyoD-tdTomato fluorescence intensity in MuSCs, cultured in a 96-well-plate, was quantitatively evaluated using an all-in-one fluorescence microscope BX-X810 equipped with an image cytometer module (BZ-H4XI, KEYENCE, Osaka, Japan).

#### Flow cytometry analysis and cell sorting

MACS-isolated MuSCs cultured for 4–5 days were detached using 0.25% trypsin/EDTA, followed by centrifugation and resuspended in 2% FBS/PBS. The cells were incubated with APC-conjugated-a7-integrin antibody (FAB3518A, R&D Systems) for 30 min on ice, followed by the addition of propidium iodide (PI) at a 1:500 (v/v) ratio. The cells were either analyzed using a flow cytometer (CytoFLEX S; Beckman Coulter, Brea, CA, USA) or sorted using a MoFlo XDP flow cytometer (Beckman Coulter). Debris and dead cells were excluded by forward scatter, side scatter, and propidium iodide (PI) gating. Gates were defined based on isotype or WT control fluorescence. Data were analyzed using FlowJo software ver. 10.7.1 (BD Biosciences, NJ, USA).

#### RNA isolation and RT-qPCR

Total RNA was extracted from the cells using TRIzol reagent (Life Technology) according to the manufacturer’s instructions. cDNA was synthesized using a ReverTra Ace Kit with genomic DNA remover (Toyobo). RT-qPCR was performed using Thunderbird SYBR Green in a 7500 Fast Real-Time PCR System (Applied Biosystems). RT-PCR primer sequences are listed in Table S3. Quantitative data were obtained in triplicate within a single experiment. All data were normalized to an internal control (*TATA-box binding protein, TBP*).

#### RNA-seq analysis

RNA was extracted from cultured cells using the TRIzoL reagent as indicated in the instructions (Thermo Scientific). Sequencing libraries of mRNA from three technical replicates were prepared using the NEBNext Ultra II RNA Library Prep Kit for IIumina and the NEBNext Poly(A) mRNA Magnetic Isolation Module (New England Biolabs, pwich, MA, USA) with 187–800 ng of total RNAs, according to the manufacturer’s instructions. The concentration of the library was adjusted to 1 nM and subsequently subjected to denaturation and neutralization. The mRNA-seq libraries were sequenced using NextSeq500/550 v2.5 (75 Cycles) Kits (Illumina, San Diego, CA, USA) in the NextSeq 500 System (Illumina). Sequencing was performed using paired-end reads of 36 bases. The basic information of the NGS run data was assessed using CLC Genomics Workbench 20.0.3 software (QIAGEN, Hilden, Germany). The read number was approximately 28–31 million per sample, as paired-end reads. Transcripts per million were also obtained using an algorithm “RNA-seq Analysis” as the default setting in CLC Genomics Workbench 20.0.3 software. Genes with zero counts in any sample were excluded, and differential expression was analyzed using integrated differential expression and pathway analysis.^58^ DEGs were extracted among conditions (low vs. high, QSCs vs. high) with a false discovery rate (FDR) of p < 0.05. Metascape was used for gene ontology analysis, with a p-value <0.01.^59^ A PCA plot was constructed using Python software.

### Quantification and statistical analysis

Values are reported as mean ± standard error of the mean (s.e.m). Statistical analyses were performed using unpaired Student’s t-tests or non-repeated measures analysis of variance (ANOVA), followed by the Bonferroni post hoc test using GraphPad Prism (GraphPad Software, Inc., San Diego, CA). p-values for statistical significance are indicated as ∗p < 0.05, ∗∗p < 0.01, ∗∗∗p < 0.001, or not significant (ns).

## Data Availability

• RNA-seq data have been deposited in the NCBI Gene Expression Omnibus (GEO). The accession number is listed in the key resources table. • This paper does not report original code. • Any additional information required to reanalyze the data repotted in this paper is available from the lead contact upon request.

## Supplemental information

Document S1. Figures S1–S9 and Tables S3

Table S1. RNA sequencing results from the QSCs, MyoD-tdTomato^low^, and MyoD-tdTomato^high^, related to Figure 6

Table S2. Differentially Expressed Genes and Gene Ontology analysis in the QSCs, MyoD-tdTomato^low^, and MyoD-tdTomato^high^, related to Figure 6 **(Tab1)** List of genes commonly upregulated in MyoD-tdTomato^low^ and QSCs when compared to MyoD-tdTomato^high^, related to Figure 6J. **(Tab2)** List of genes commonly downregulated in MyoD-tdTomato^low^ and QSCs compared to MyoD-tdTomato^high^, related to Figure 6K. **(Tab 3)** List of genes specifically upregulated in MyoD-tdTomato^low^ when compared to MyoD-tdTomato^high^, related to Figure 6L. **(Tab 4)** List of genes specifically downregulated in MyoD-tdTomato^low^ when compared to MyoD-tdTomato^high^, related to Figure 6M.
