# Supplementary material for: Generation of a MyoD knock-in reporter mouse line to study muscle stem cell dynamics and heterogeneity
Source: iScience. 2023 Apr 8;26(5):106592. doi: 10.1016/j.isci.2023.106592 (PMC10214404; doi:10.1016/j.isci.2023.106592)
Supplement: Document S1. Figures S1–S9 and Tables S3 [file mmc1.pdf]

iScience, Volume *26*

Supplemental information

Generation of a MyoD knock-in reporter mouse line to study muscle stem cell dynamics and heterogeneity

Ryo Fujita, Seiya Mizuno, Taketaro Sadahiro, Takuto Hayashi, Takehito Sugasawa, Fumihiro Sugiyama, Yusuke Ono, Satoru Takahashi, and Masaki Ieda

Supplementary Data

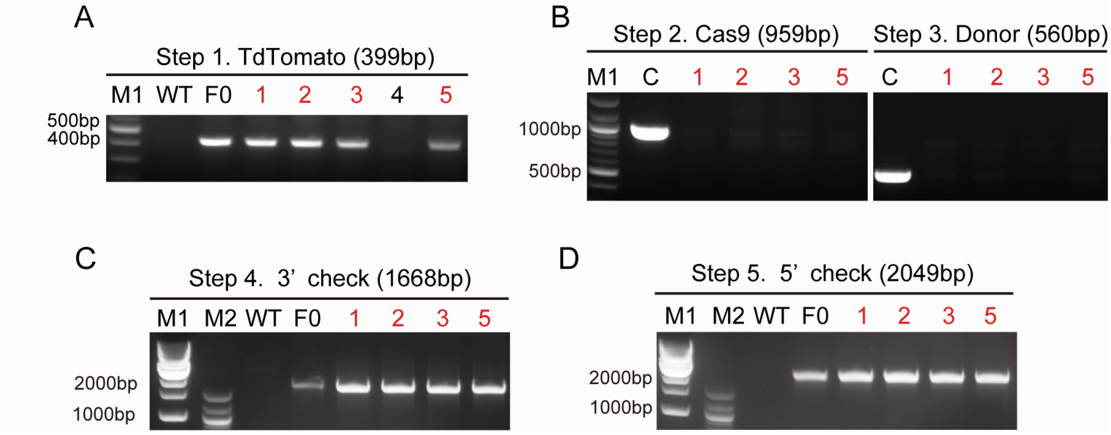

Figure S1. PCR analysis of F1 MyoD knock-in (KI) mice, related to Figure 1.

(A-D) Genomic DNA analysis of F1 MyoD$^{KI/+}$ and WT mouse tails using PCR. The primer sets used for steps 1, 4, and 5 as shown in Fig. 1B. Five F1 mice (1–5) and their parents (female WT and male F0 #10) were subjected to PCR analysis. The expected size of each PCR amplicon is indicated in parentheses. M1, 1 kb DNA ladder; M2, 100 bp DNA ladder; C, Cas9-amp control.

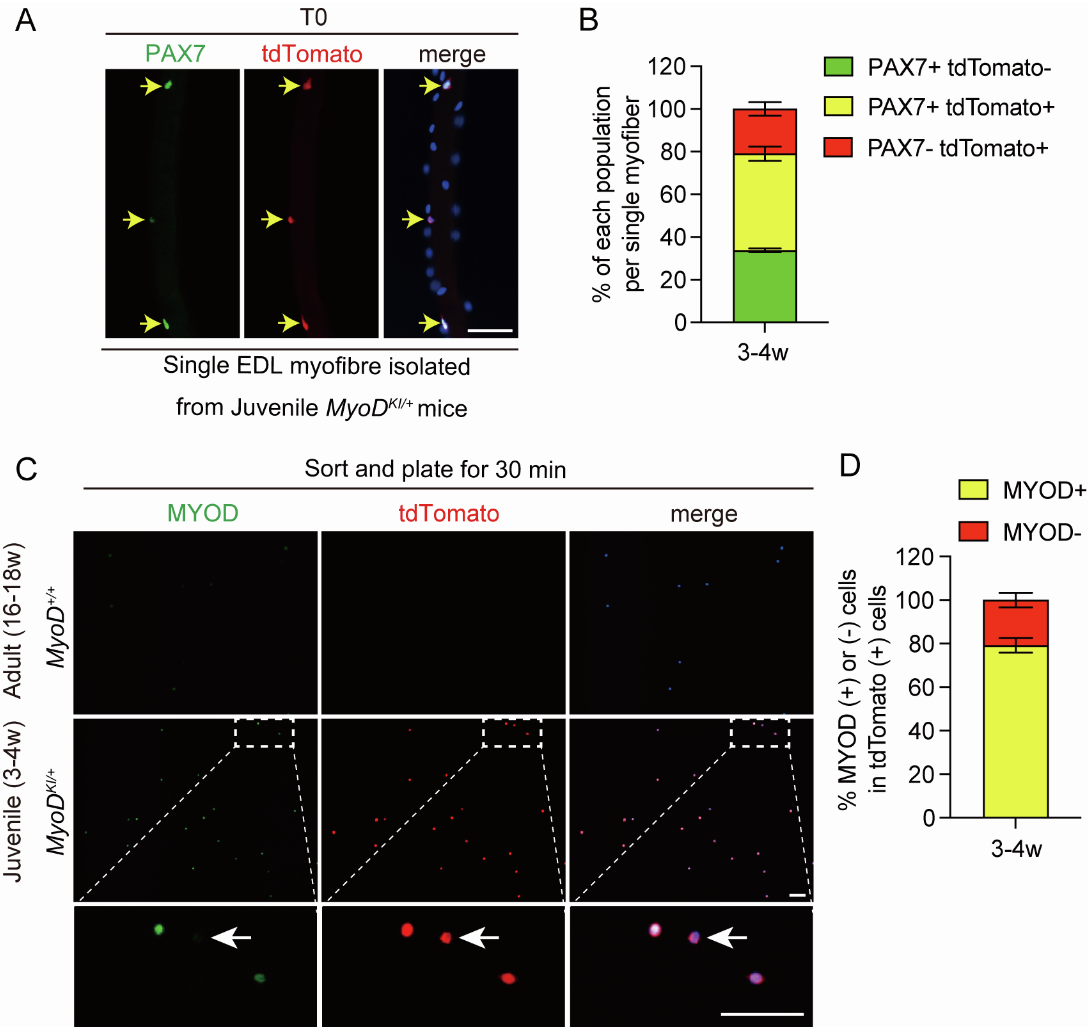

Figure S2. Analysis of MyoD-tdTomato fluorescence in muscle stem cells (MuSCs) from juvenile *MyoD*$^{KI/+}$ mice *in vivo*, related to Figure 2.

(A) Immunofluorescence of PAX7 (green) and tdTomato (red) on freshly isolated EDL myofibers (T0) from 3–4-week-old *MyoD*$^{KI/+}$ mice. Nuclei stained with DAPI. Yellow arrows indicate PAX7 (+) and tdTomato (+) cells on EDL myofibers. Scale bar: 50 µm. **(B)** The proportion of PAX7 (+), tdTomato (-) (green), PAX7 (+), tdTomato (+) (yellow), and PAX7 (-), tdTomato (+) (red) cells in **A**. **(C)** tdTomato (+) cells sorted from 3–4-week-old *MyoD*$^{KI/+}$ mice using flow cytometer, and these cells are re-plated for 30 min to perform immunofluorescence analysis with antibodies against MYOD (green) and tdTomato (red). MuSCs from 16–18-week-old *MyoD*$^{+/+}$ mice was used as a negative control for MYOD and tdTomato staining. The area in white dotted boxes is shown at higher magnification. White arrows indicate MYOD (-), tdTomato (+) cells. Scale bar: 50 µm. **(D)** The proportion of MYOD (+) or MYOD (-) cells in sorted tdTomato (+) cells in **C** (n = 4 mice). All data are represented as the mean ± standard error of the mean (s.e.m.).

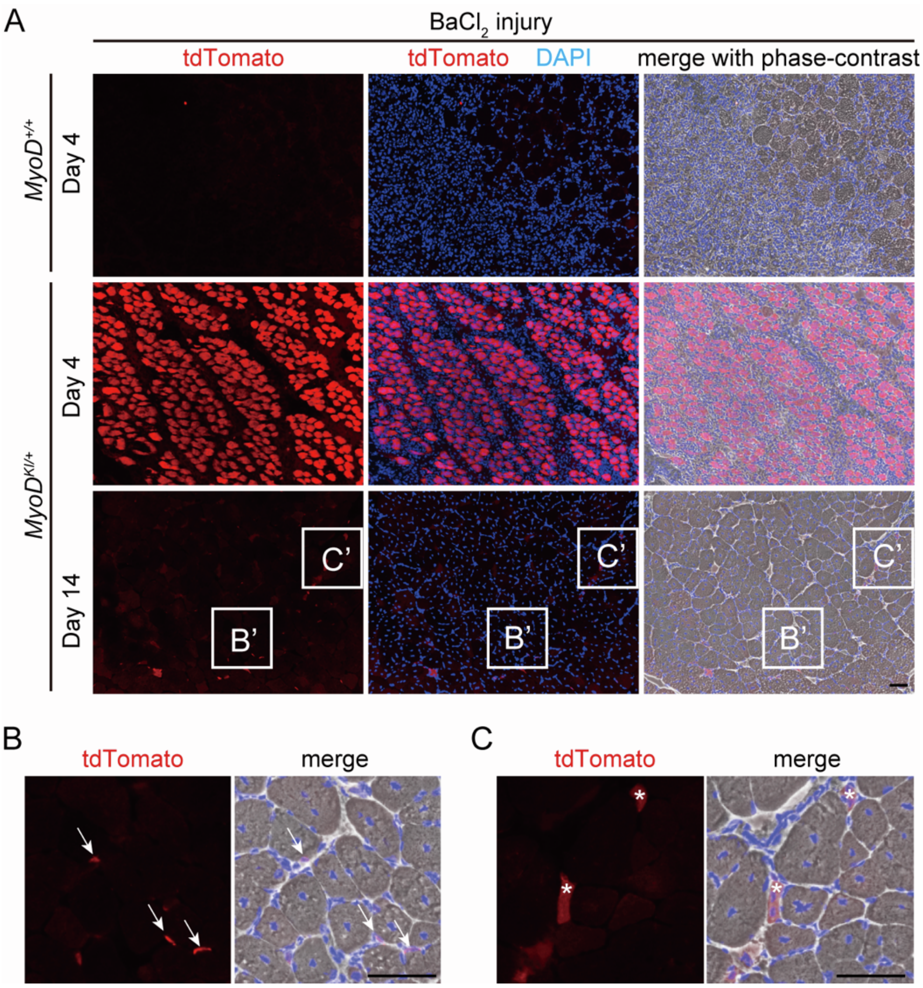

Figure S3. Analysis of MyoD-tdTomato fluorescence in damaged and regenerated myofibers following $BaCl_2$ injury, related to Figure 2 and 3.

(A-C) TA muscle cross-sections from *MyoD*$^{+/+}$ or *MyoD*$^{KI/+}$ mice at 4- or 14-day post $BaCl_2$ injury. Nuclei stained with DAPI. The regions depicted in B' and C' are shown at higher magnification in **(B)** and **(C)**, respectively. White arrows in **(B)** indicate tdTomato+ cells associated with the regenerated myofibers at day 14 post injury. White asterisks in **(C)** show tdTomato+ myofibers at day 14 post injury. Scale bars: 50 µm.

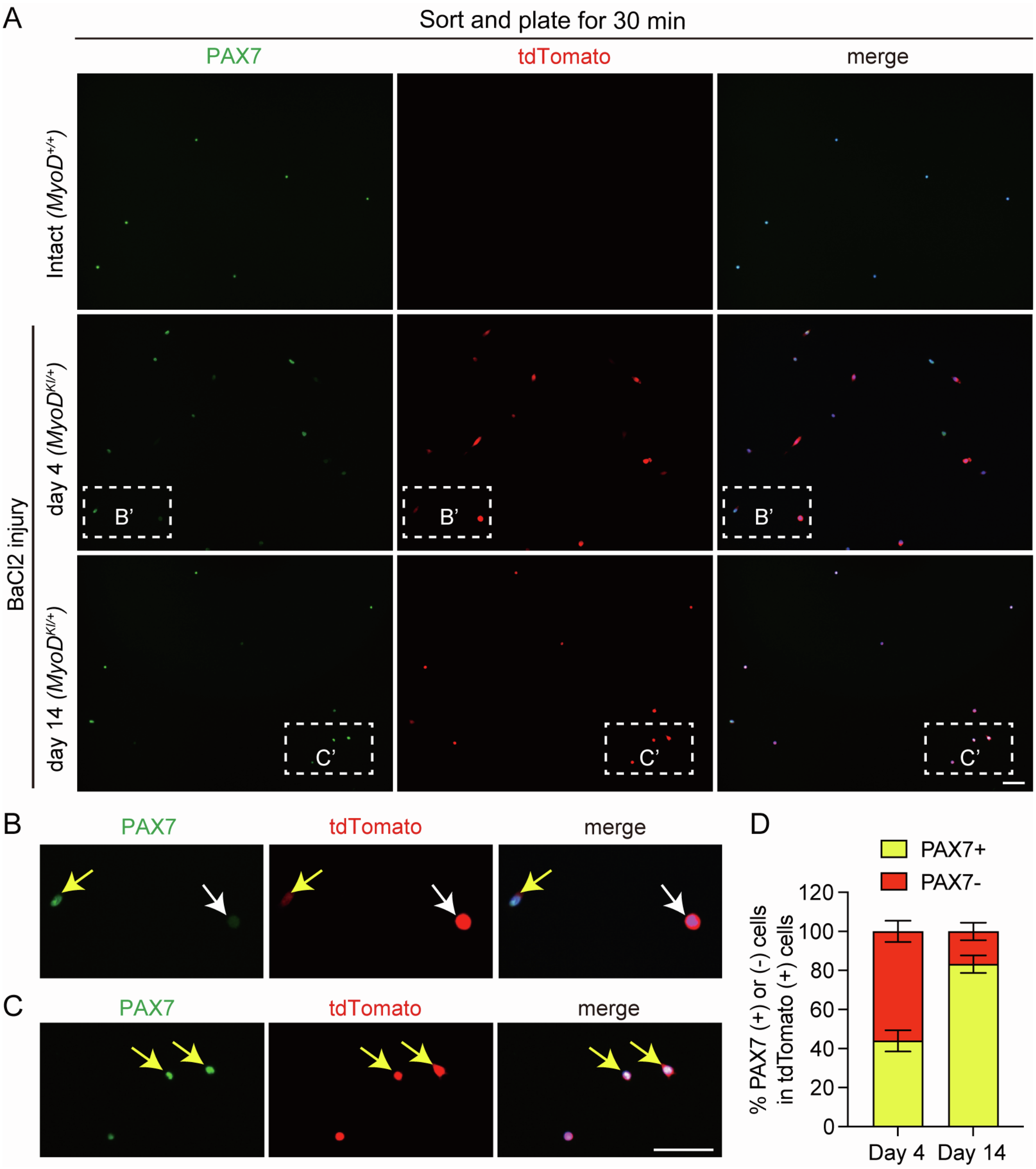

Figure S4. Analysis of MyoD-tdTomato fluorescence and PAX7 expression in the MuSCs isolated from damaged and regenerated muscles following $BaCl_2$ injury, related to Figure 2.

(A-C) tdTomato (+) cells isolated from *MyoD*$^{KI/+}$ mice at 4- and 14-days post $BaCl_2$ injury using flow cytometer, and these cells are re-plated for 30 min to perform immunofluorescence analysis with antibodies against PAX7 (green) and tdTomato (red). The regions depicted in B' and C' are shown at a higher magnification in **(B)** and **(C)**, respectively. Yellow arrows indicate PAX7 (+), tdTomato (+) cells. White arrows indicate PAX7 (-) tdTomato+ cells. Scale bars: 50 μm. **(D)** The proportion of PAX7 (+) or PAX7 (-) cells in sorted tdTomato (+) cells in **A** (n = 4 mice). All data are represented as the mean ± s.e.m.

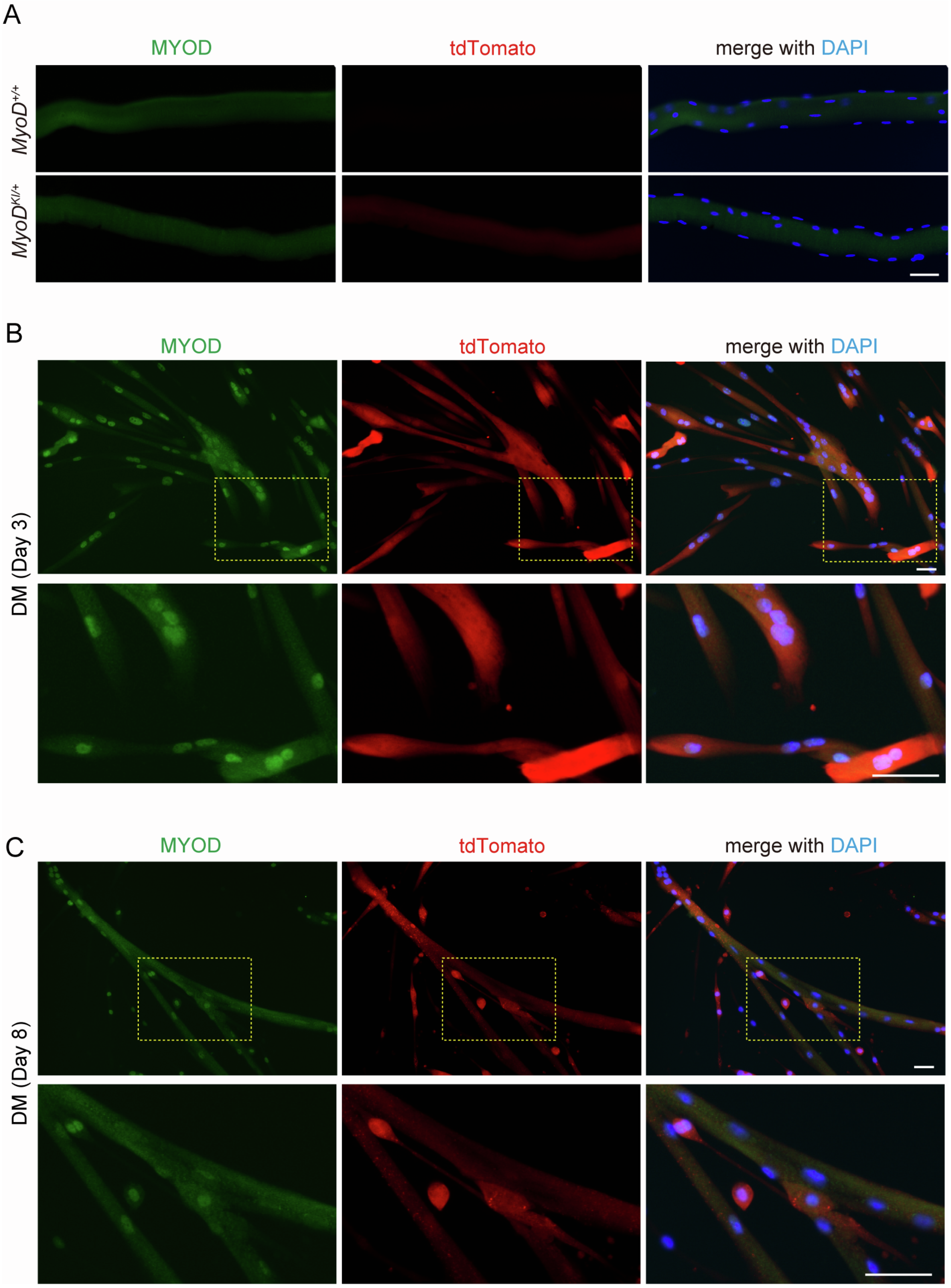

Figure S5. Analysis of MyoD-tdTomato fluorescence in differentiating myotubes *in vitro*, related to Figure 2 and 3.

(A-B) Immunofluorescence of MYOD (green) and tdTomato (red) on primary myotubes from *MyoD*$^{KI/+}$ mice. Nuclei stained with DAPI. MuSCs from *MyoD*$^{KI/+}$ mice were cultured in grown medium for 4 days, media was replaced with differentiation media and the cells were cultured for **(A)** 3 days **(B)** or 8 days. Scale bars: 50 μm.

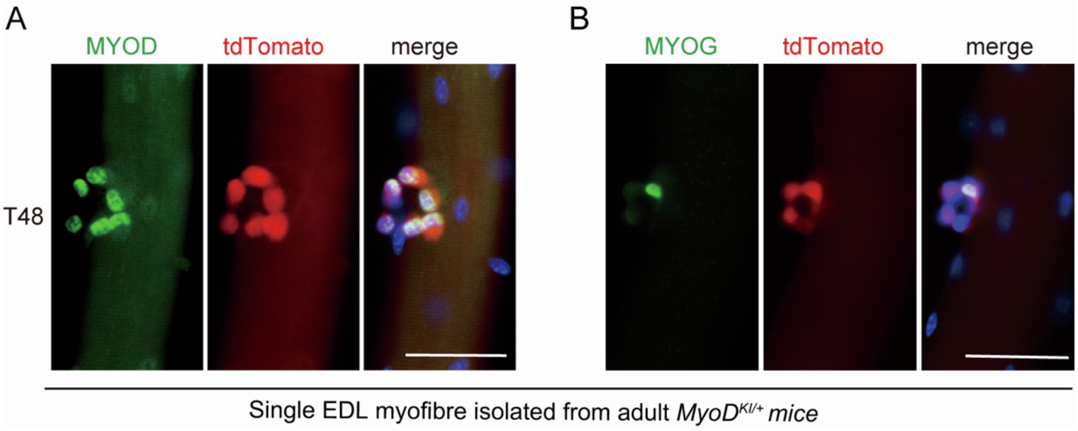

Figure S6. Immunofluorescence analysis of MyoD-tdTomato with MYOD or MYOGENIN antibodies on single EDL myofibers after 48 h culture, related to Figure 3.

(A-B) Immunofluorescence of MYOD **(A)** or MYOGENIN **(B)** and tdTomato on single EDL myofibers cultured for 48 h (T48) from 15–16-week-old *MyoD*$^{KI/+}$ mice. Nuclei stained with DAPI. Scale bar: 50 µm.

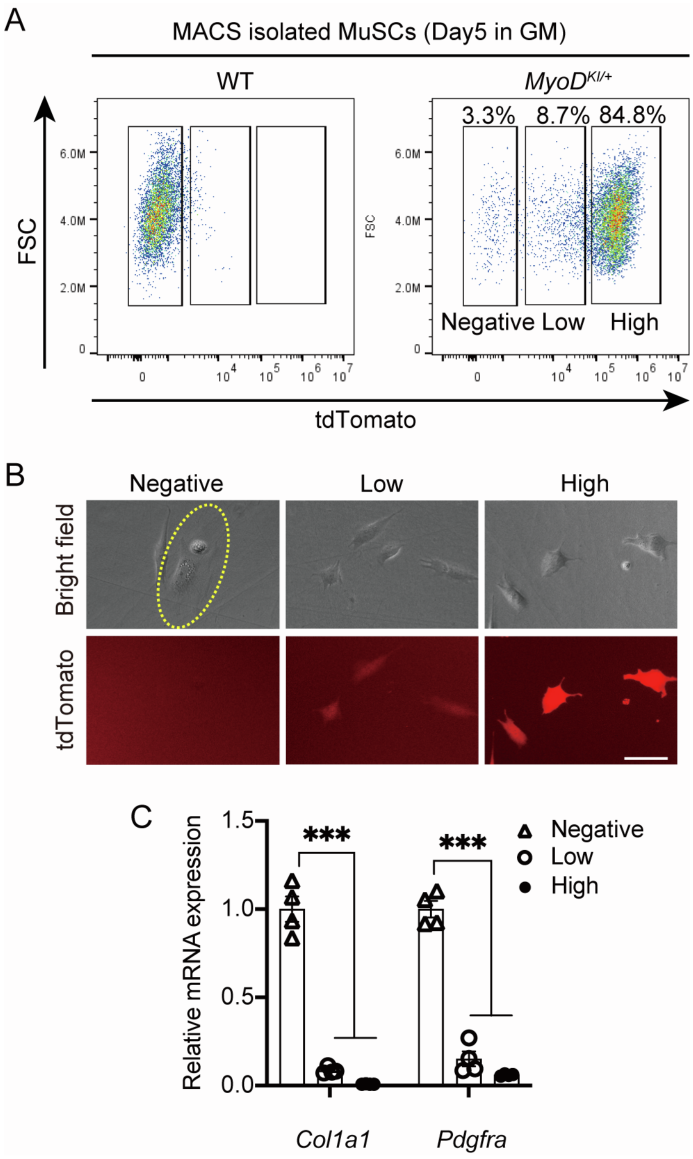

Figure S7. MyoD-tdTomato fluorescence was restricted to the myogenic progenitors, related to Figure 3, 5, and 6.

(A) Representative flow cytometry plot of cultured MuSCs isolated from $MyoD^{+/+}$ (WT) and $MyoD^{KI/+}$ mice. The negative gate was defined based on WT MuSC analysis. **(B)** Cultured MACS-isolated MuSCs from $MyoD^{KI/+}$ mice were gated into MyoD-tdTomato^{negative}, MyoD-tdTomato^{low}, and MyoD-tdTomato^{high} groups and sorted using flow cytometry. These cells were re-plated for 4 h to allow attachment to the dishes. The MyoD-tdTomato^{negative} population contains fibroblastic cells with a large cytosol, as indicated by the yellow circle. **(C)** Relative expression of *Col1a1 and Pdgfra*, markers for fibroblasts, determined by RT-qPCR, in MyoD-tdTomato^{negative}, MyoD-tdTomato^{low} and MyoD-tdTomato^{high} sorted using flow cytometry (n = 4 mice). All data are represented as the mean ± s.e.m. ***$P < 0.001$.

Figure S8. Full Sequence of 3'-MyoD-tdTomato PCR product amplified in Step 4, related to Figure 1.

tdTomato ***STOP codon*** *MyoD exon3*

GAGCGCGTGATGAACTTCGAGGACGGCGGTCTGGTGACCGTGACCCAGGACTCCTCCCTGCAGGACGGCACGCTGATCTAC
AAGGTGAAGATGCGCGGCACCAACTTCCCCCCCGACGGCCCCGTAATGCAGAAGAAGACCATGGGCTGGGAGGCCTCCAC
CGAGCGCCTGTACCCCCGCGACGGCGTGCTGAAGGGCGAGATCCACCAGGCCCTGAAGCTGAAGGACGGCGGCCACTACC
TGGTGGAGTTCAAGACCATCTACATGGCCAAGAAGCCCGTGCAACTGCCCGGCTACTACTACGTGGACACCAAGCTGGACA
TCACCTCCCACAACGAGGACTACACCATCGTGGAACAGTACGAGCGCTCCGAGGGCCGCCACCACCTGTTCCTGTACGGCAT
GGACGAGCTGTACAAGTAAGAATTC**TGA**GAGATCGACTGCAGCAGCAGAGGGCGCACCACCGTAGGCACTCCTGGGGATG
GTGTCCCTGGTTCTTCACGCCCAAAAGATGAAGCTTAAATGACACTCTTCCCAACTGTCCTTTCGAAGCCGTTCTTCCAGAGG
GAAGGGAAGAGCAGAAGTCTGTCCTAGATCCAGCCCCAAAGAAAGGACATAGTCCTTTTTGTTGTTGTTGTTGTAGTCCTTC
AGTTGTTTGTTTGTTTTTTCATGCGGCTCACAGCGAAGGCCACTTGCACTCTGGCTGCACCTCACTGGGCCAGAGCTGATCCT
TGAGTGGCCAGGCGCTCTTCCTTTCCTCATAGCACAGGGGTGAGCCTTGCACACCTAAGCCCTGCCCTCCACATCCTTTTGTT
TGTCACTTTCTGGAGCCCTCCTGGCACCCACTTTTCCCCACAGCTTGCGGAGGCCACTCAGGTCTCAGGTGTAACAGGTGTAA
CCATACCCCACTCTCCCCCTTCCCGCGGTTCAGGACCACTTATTTTTTTATATAAGACTTTTGTAATCTATTCGTGTAAATAAGA
GTTGCTTGGCCAGAGCGGGAGCCCCTTGGGCTATATTTATCTCCCAGGCATGCTGTGTAGTGCAACAAAAACTTTGTATGTT
TATTCCTCAAGCGGGCGAGCCTCGAGGCTCGCTCGCTCAGGTGTTGGAAATAAAGACGCTAATTTATACAAAGTGGCTCTG
GCTTTTCCTAAGGGGATCAGAAAGAAACTCTACGAACTGGGCGGGCTGTCTCGCAGCGACCCCTGTAGGTGGCAGAAGGG
TAGCACGGAGGCTGGGTAGTGCTGGGTAATGAAGAAGGGCTGGCAGACCTCCAGCTGTAGGGAATTCCCCAGGCCTTGCC
TGCCGCACCCAAGAAAACCAGTGGCTCCGGTGGAAAGATGCACGTAGGTTGATGTTCGTTTAAAAATACAAAAAGCCAGAT
GTGGCGGCTCATCCCTGTAATCTCAACATATTTGAGAAGCCGAGGCAGGAGGATGGAGGCAAGCTGGAGGCCACCCTGGC
GATAAAGACATAACCTCTTTAAAATACAAAATTAAAAGTTGGTGTTAAGGAGGTGCTCAGGTGATAGAGCACTTGCCTAGC
ATGCACAAAATCCCAGGCTCAGTCCCCAGCACCATATCAACCTGTCATGGGGGACACAGCTGTAGTTTCAAGGCCCGGAAG
GNNNNNCCAAGGAAAAAA

Figure S9. Full sequence of 5'-MyoD-tdTomato PCR product amplified in Step 5, related to Figure 1.

MyoD exon1-3 *P2A* *tdTomato*

TCCTGCGCAACGCCATCCGCTACATCGAAGGTCTGCAGGCTCTGCTGCGCGACCAGGACGCCGCGCCCCCTGGCGCCGCTG
CCTTCTACGCACCTGGACCGCTGCCCCCAGGCCGTGGCAGCGAGCACTACAGTGGCGACTCAGATGCATCCAGCCCGCGCT
CCAACTGCTCTGATGGCATGGTAAGGCGGGGGGCTCAGGAGGATGAGCAATGGAGGCGGCGCCTGGGGTATCTGCAACA
GGTTTCCGAGGCCCTTGGGGTGGGGGTGTCCCTTATACCTAGATGCTCCTGGCATCTGACACTGGAGTCGCTTTGGAGACCC
AGGGCATCTATGATTCTGCCGATTGGGGGTGGAACACTGCTGCGCAGACCCCGGGATATGCTTTTCCTTCTCATTATTACCCT
AATGCAGATTATTGTTCCTGAGTGACTGTCCACTCTCAGTTTGGCCCCGCATGCGACAGCTTCCAGTGTGTGGCTGGCTCCTA
CCACCTGGGGCTGACCCAGTCCTGGAACCAGCAGCTGAGACTAAGGGAGTGAGGGAGGGGTGATGACAAGGAGTGTTGC
TTGAGACCCACTCGGGCCCTGTAGACCTAACTCTGTTATCCTTGCTATTCGCAGATGGATTACAGCGGCCCCCCAAGCGGCCC
CCGGCGGCAGAATGGCTACGACACCGCCTACTACAGTGAGGCGGCGCGCGGTGCGTATTCTCAGCTGTTCCCAGCTAGCAG
GCCTTTATCGGCCTTCTGTATCCCCCTTGAAACTTTCCTCGCTCCCTAGGCTTAGTATCCTCTTCCTGCCTCCACCACATACATA
CCCGTACCTTGGGATGGCGGGGGGGGGGGAGGCTGGGGGGGGAGCATTGGGGGAGGGGACAAAGAACTATGATGCAC
ACCTTCCTCTCCTTTCTCCTTCCAGTCTAGCAAGTCCTCAGTTTCCCTTTTGCTACAAAGCTCCGTGCCTATGGGCAGGAGACTT
GAGAAGGGCCGCAAGTTTGGATTACTAACCTTCCACTCCCCTCACAGAGTCCAGGCCAGGGAAGAGTGCGGCTGTGTCGAG
CCTCGACTGCCTGTCCAGCATAGTGGAGCGCATCTCCACAGACAGCCCCGCTGCGCCTGCGCTGCTTTTGGCAGATGCACCA
CCAGAGTCGCCTCCGGGTCCGCCAGAGGGGGCATCCCTAAGCGACACAGAACAGGGAACCCAGACCCCGTCTCCCGACGC
CGCCCCTCAGTGTCCTGCAGGCTCAAATCCTAACGCGATTTATCAGGTGCTTAAGCTTGGAAGCGGAGCCACCAACTTCTCCC
TGCTGAAGCAGGCCGGCGACGTGGAGGAGAACCCCGGCCCCATGGTGAGCAAGGGCGAGGAGGTCATCAAAGAGTTCAT
GCGCTTCAAGGTGCGCATGGAGGGCTCCATGAACGGCCACGAGTTCGAGATCGAGGGCGAGGGCGAGGGCCGCCCCTAC
GAGGGCACCCAGACCGCCAAGCTGAAGGTGACCAAGGGCGGCCCCCTGCCCTTCGCCTGGGACATCCTGTCCCCCCAGTTC
ATGTACGGCTCCAAGGCGTACGTGAAGCACCCCGCCGACATCCCCGATTACAAGAAGCTGTCCTTCCCCGAGGGCTTCAAGT
GGGAGCGCGTGATGAACTTCGAGGACGGCGGTCTGGTGACCGTGACCCAGGACTCCTCCCTGCAGGACGGCACGCTGATC
TACAAGGTGAAGATGCGCGGCACCAACTTCCCCCCCGACGGCCCCGTAATGCAGAAGAAGACCATGGGCTGGGAGGCCTC
CACCGAGCGCCTGTACCCCCGCGACGGCGTGCTGAAGGGCGAGATCCACCAGGCCCTGAAGCTGAAGGACGGCGGCCACT
ACCTGGTGGAGTTCAAGACCATCTACATGGCCAAGAAGCCCGTGCAACTGCCCGGCTACT

Table S3. List of primer sequences used in this study, related to STAR Methods.

**MyoD-KI screening**	**Orientation**	**Sequence (5' to 3')**
tdTomato (Step 1)	Fw	CAGTTCATGTACGGCTCCAA
	Rv	GAGGTGATGTCCAGCTTGGT
Cas9 (Step 2)	Fw	AGTTCATCAAGCCCATCCTG
	Rv	GAAGTTTCTGTTGGCGAAGC
Amp (Step 3)	Fw	TTGCCGGGAAGCTAGAGTAA
	Rv	TTTGCCTTCCTGTTTTTGCT
Myod1 3' (Step 4)	Fw	ATCCCCGATTACAAGAAGCTGTCCTTC
	Rv	TTTTCCTGTGTTACCTTCCGGGCCTTGA
Myod1 5' (Step 5)	Fw	AAGTGAATGAGGCCTTCGAGACGCTCAA
	Rv	GCTCGTACTGTTCCACGATGGTGTAGTC
**Routine genotyping**	**Orientation**	**Sequence (5' to 3')**
tdTomato (Step 1)	Rv	GAGGTGATGTCCAGCTTGGT
Myod 1	Fw	ATGGGCAGGAGACTTGAGAA
	Rv	CGAAAGGACAGTTGGGAAGA
**RT-qPCR**	**Orientation**	**Sequence (5' to 3')**
Pax7	Fw	CTCAGTGAGTTCGATTAGCCG
	Rv	AGACGGTTCCCT TTGTCGC
Myod1	Fw	CCCCGGCGGCAGAATGGCTACG
	Rv	GGTCTGGGTTCCCTGTTCTGTGT
Myogenin	Fw	CAACCAGGAGGAGCGCGATCTCCG
	Rv	AGGCGCTGTGGGAGTT GCATTCACT
tdTomato	Fw	ACCGCCAAGCTGAAGGTGAC
	Rv	TTGAAGCCCTCGGGGAAGGA
Col1a1	Fw	CCTCAGGGTATTGCTGGACAAC
	Rv	CAGAAGGACCTTGTTTGCCAGG
Pdgfra	Fw	GCAGTTGCCTTACGACTCCAGA
	Rv	GGTTTGAGCATCTTCACAGCCAC
TATA-box binding protein (TBP)	Fw	CAGATGTGCGTCAGGCGTTC
	Rv	TAGTGATGCTGGGCACTGCG
**Sequence analysis**	**Orientation**	**Sequence (5' to 3')**
Myod1 3' (Step 4)	Fw1	ATCCCCGATTACAAGAAGCTGTC
	Fw2	AGAGGGAAGGGAAGAGCAGAA
	Fw3	TCGCTCGCTCAGGTGTTGG

Myod1 5′ (Step 5)	Fw1	AAGTGAATGAGGCCTTCGAGAC
	Fw2	AGGGGTGATGACAAGGAG
	Rv1	GCTCGTACTGTTCCACGATGGT
	Rv2	AAGCGCATGAACTCTTTG
